# Gene expression and biological processes influenced by deletion of *Stat3 *in pulmonary type II epithelial cells

**DOI:** 10.1186/1471-2164-8-455

**Published:** 2007-12-10

**Authors:** Yan Xu, Machiko Ikegami, Yanhua Wang, Yohei Matsuzaki, Jeffrey A Whitsett

**Affiliations:** 1 Division of Pulmonary Biology, Cincinnati Children's Hospital Medical Center, Department of Pediatrics, University of Cincinnati College of Medicine, 3333 Burnet Avenue, Cincinnati, OH, USA

## Abstract

**Background:**

The signal transducer and activator of transcription 3 (STAT3) mediates gene expression in response to numerous growth factors and cytokines, playing an important role in many cellular processes. To better understand the molecular mechanisms by which *Stat3 *influences gene expression in the lung, the effect of pulmonary epithelial cell specific deletion of *Stat3 *on genome wide mRNA expression profiling was assessed. Differentially expressed genes were identified from Affymetrix Murine GeneChips analysis and subjected to gene ontology classification, promoter analysis, pathway mapping and literature mining.

**Results:**

Total of 791 mRNAs were significantly increased and 314 mRNAs were decreased in response to the deletion of *Stat3*^Δ/Δ ^in the lung. STAT is the most enriched cis-elements in the promoter regions of those differentially expressed genes. Deletion of *Stat3 *induced genes influencing protein metabolism, transport, chemotaxis and apoptosis and decreased the expression of genes mediating lipid synthesis and metabolism. Expression of *Srebf1 *and *2*, genes encoding key regulators of fatty acid and steroid biosynthesis, was decreased in type II cells from the *Stat3*^Δ/Δ ^mice, consistent with the observation that lung surfactant phospholipids content was decreased. *Stat3 *influenced both pro- and anti-apoptotic pathways that determine cell death or survival. *Akt*, a potential transcriptional target of *Stat3*, was identified as an important participant in *Stat3 *mediated pathways including Jak-Stat signaling, apoptosis, Mapk signaling, cholesterol and fatty acid biosynthesis.

**Conclusion:**

Deletion of *Stat3 *from type II epithelial cells altered the expression of genes regulating diverse cellular processes, including cell growth, apoptosis and lipid metabolism. Pathway analysis indicates that STAT3 regulates cellular homeostasis through a complex regulatory network that likely enhances alveolar epithelial cell survival and surfactant/lipid synthesis, necessary for the protection of the lung during injury.

## Background

The signal transducers and activators of transcription (STATs) were initially identified as a family of latent cytoplasmic transcription factors that are activated by various cytokines, growth factors and other stimuli, and phosphorylated by many protein kinases [1-4]. In response to various stimuli, STAT family members are phosphorylated by receptor associated kinases, form homo- or heterodimers and are translocated to the cell nucleus where they activate transcription [5]. Recent studies also support the role of unphosphorylated STAT3 that accumulates in nucleus and activates transcription by binding to NFkappaB [6]. STAT3 regulates a variety of biological processes, functioning at both transcriptional and non-transcriptional levels to influence cell growth, survival and metabolism. Its capacity to induce cell transformation and tumorigenesis makes it a potential therapeutic target for various cancers [7,8].

Systemic deletion of *Stat3 *is embryonic lethal in the mouse, indicating its important role in embryogenesis [9]. Biological roles of STAT3 in various organs and cells have been studied *in vitro *as well as cell specific deletion in the mouse *in vivo*. The biological consequences of *Stat3 *deletion are surprisingly diverse and sometimes contradictory. For example, cardiomyocyte-specific STAT3 deficiency caused cardiac fibrosis and heart dysfunction with advanced age [10]. Hepatic cell specific deletion of *Stat3 *caused insulin resistance associated with increased expression of gluconeogenic genes [11]. Conditional ablation of *Stat3 *in respiratory epithelium of the mouse (*Stat3*^Δ/Δ ^mice) did not alter lung morphogenesis or function but enhanced susceptibility to hyperoxia and adenoviral induced lung injury whereas overexpression of Stat3C (a constitutive active form of STAT3) in pulmonary epithelium protects against hyperoxic lung injury, suggesting that STAT3 is required for the maintenance of surfactant homeostasis and lung function during injury [12-14]. While STAT3 has been proposed as an anti-apoptotic protein through the induction of survival genes such as Bcl2-like 1(*Bcl-X) and *B-cell leukemia/lymphoma 2 *(Bcl-2)*, STAT3 also exerts pro-apoptotic effect through the regulation of insulin-like growth factor binding protein 5 (IGFBP-5) to modulate mammary epithelial apoptosis [4,15]. STAT3 is abundantly and ubiquitously expressed in many tissues and distributed between the cell cytosol and nucleus. A direct effect of non-phosphorylated, cytoplasmic STAT3 on cell motility was reported recently through direct protein-protein interactions [16], indicating a non-transcriptional function of STAT3.

The functions of STAT3 vary in different cellular and physiologic contexts, influencing diverse gene targets by interaction with other proteins and genes. The diversity of STAT3 functions indicate that STAT3 is involved in complex genetic networks to maintain cellular homeostasis rather serving a singular role in acute phase responses as initially defined. In the present study, we sought to systemically study the role of STAT3 in pulmonary epithelial cell homeostasis. Using knowledge based gene expression profiling approaches and a conditional system that selectively deleted *Stat3 *in the respiratory epithelium; we identified a large STAT3-dependent network that influences a wide variety of biological processes in type II alveolar cells in the lung.

## Results and Discussion

### Identification of differentially expressed genes in alveolar type II epithelial cells from Stat3^Δ/Δ ^mice

In previous studies Stat3 mRNA and protein expression were markedly reduced in Type II cells isolated from *Stat3*^Δ/Δ ^mice, being less than 10% of control levels [12]. To identify the RNAs influenced by the conditional deletion of *Stat3*^Δ/Δ^, RNAs isolated from alveolar epithelial type II cells of control and *Stat3*^Δ/Δ ^mice were compared using Affymetrix murine genome MOE430 gene chips. The complete dataset can be found at Gene Expression Omnibus (GEO) [17]; Accession no. GSE6846. Total of 1105 genes were identified as significantly altered using the criteria described in Method. Among them, 791 mRNAs were increased and 314 mRNAs were decreased in response to the deletion of *Stat3*^Δ/Δ ^in the lung (Additional file 1). Changes in mRNA expression of a subset of genes including *Malt1, Rnt4, Reg3g, Bcl2l1, Cds2, Cdipt, Fasn, Acox2, Akt2, Gpam, Foxj1, Abca3, Srebf1, Srebf2 and Scap *were validated by real-time RT-PCR. Genes cross validated by RT-PCR are listed in Table 1 and indicated by asterisks (*) in Additional file 1.

**Table 1:** Comparison of mRNAs by RT-PCR and RNA microarray

GENE	RT-PCR	Microarray	P-Value for RT-PCR	Gene Name
Malt1	3.5	6.27	< 0.050	Mucosa associated lymphoid tissue lymphoma translocation gene 1
Rtn4	2.7	4.86	< 0.050	reticulon 4
Reg3g	-5.5	-6.02	< 0.050	regenerating islet-derived 3 gamma
Bcl2l1	-1.2	-1.54	0.053	Bcl2-like 1
Akt2	-1.5	-1.58	< 0.050	thymoma viral proto-oncogene 2
Abca3	-2.4	-1.59	< 0.050	ATP-binding cassette, sub-family A (ABC1), member 3
Scap	-1.4	-1.58	< 0.050	SREBP cleavage activating protein
Srebf1	-1.4	-1.69	< 0.050	sterol regulatory element binding factor 1
Srebf2	-1.3	-1.50	0.080	sterol regulatory element binding factor 2
Cdipt	-1.8	-1.54	< 0.001	CDP-diacylglycerol--inositol 3-phosphatidyltransferase
Fasn	-1.7	-1.57	< 0.001	fatty acid synthase
Acox2	-1.5	-1.67	< 0.001	acyl-Coenzyme A oxidase 2
Cds2	-2.1	-1.78	< 0.001	CDP-diacylglycerol synthase (phosphatidate cytidylyltransferase)
Gpam	-1.5	-1.54	< 0.001	glycerol-3-phosphate acyltransferase

Note: Fold change of mRNA in *Stat3*^Δ/Δ ^mice to control is shown.

### Functional classification of differentially expressed genes revealed the dysregulation of various biological processes in type II epithelial cells from Stat3^Δ/Δ ^mice

Differentially expressed genes were classified according to Gene Ontology (GO) classification on Biological Process. The Fisher Exact Test was used to calculate the probability of each category that was overrepresented in the selected list using the entire MOE430 mouse genome as reference dataset. Deletion of *Stat3 *from type II cells significantly induced the genes involved in protein metabolism, protein transport, chemotaxis and apoptosis and decreased the expression of genes in lipid synthesis and metabolism (Table 2).

**Table 2:** Functional Classification of Genes Differentially Expressed in *Stat3*^Δ/Δ ^mice

GO Classification of Genes Up-regulated in *Stat3*^Δ/Δ ^mice			
*Term*	*Count*	*%*	*PValue*

Biopolymer modification	115	13.94	7.30E-13
Cellular protein metabolism	164	19.88	5.83E-10
Protein transport	52	6.30	2.49E-07
Phosphate metabolism	62	7.52	3.31E-07
Chemotaxis	17	2.06	1.38E-06
Protein kinase cascade	24	2.91	6.83E-06
Apoptosis	40	4.85	1.69E-05
Regulation of apoptosis	29	3.52	4.93E-05
Positive regulation of cellular metabolism	25	3.03	9.95E-05
Cell migration	19	2.30	2.26E-03
Transcription	106	12.85	2.64E-03

GO Classification of Genes Down-regulated in *Stat3*^Δ/Δ ^mice			

*Term*	*Count*	*%*	*PValue*

Cellular lipid metabolism	33	9.97	3.60E-13
Lipid biosynthesis	21	6.34	3.59E-11
Sterol metabolism	10	3.02	2.96E-07
Fatty acid metabolism	14	4.23	5.35E-07
Steroid metabolism	12	3.63	6.83E-06
Carboxylic acid metabolism	18	5.44	5.84E-04
Coenzyme metabolism	10	3.02	2.51E-03
Cellular carbohydrate metabolism	12	3.63	3.98E-03
Electron transport	14	4.23	2.64E-02
Phosphate metabolism	21	6.34	3.27E-02

Note: Gene Ontology Analysis was preformed using public available web-based tool DAVID [81]. Overrepresented biological processes were selected at the threshold of Fisher Exact Test P-Value ≤ 0.05 and minimum gene counts belonging to an annotation term ≥ 2%.

### Promoter analysis identified putative common regulators of the differentially expressed genes

To identify putative common transcription factors regulating the type II cell responses to *Stat3 *deletion, promoter region (-2kb to exon1) of differentially expressed genes were searched for overrepresented cis-elements using MatInspector (Genomatix) vertebrate matrix library. In compare with the sequence of random chosen gene promoters, the cis-elements significantly enriched in the promoter region of differentially expressed genes were selected based on a binomial probability calculation and their percentage frequency in our selected gene list (Adjusted p Value < 0.001 and frequency > 50 %). STAT, EGRF, AHRR, SP1F, ZF5F, E2FF, HIFF, SREB and AP2F were the most overrepresented cis-elements and may therefore mediate changes in gene expression in cells from *Stat3*^Δ/Δ ^mice (Table 3). The finding that STAT was the most enriched cis-elements indicated the identification of a sub-group of potential direct transcriptional targets of *Stat3 *in lung epithelial cells. Sterol regulatory element binding factors (*Srebf1 and 2*, known as important transcriptional regulators of fatty acid and steroid biosynthesis), were significantly decreased in *Stat3*^Δ/Δ ^mice. The SREB binding site was overrepresented in differentially expressed genes indicating that it is a potential regulator of the lipid metabolism pathways altered in *Stat3*^Δ/Δ ^mice. Other significantly enriched TFBS include SP1F and HIFF. STAT3 and SP1 function cooperatively to activate the C/EBP promoter, the SP1 site being required for IL-6 induction and transactivation by STAT3 [18]. HIF1A, SP1, SMAD3 AND SMAD4 can form multifactor complex, regulating VEGF and erythropoietin gene transcription through functional cooperation and association [19-21]. Consistent with the promoter analyses and literature findings, the mRNA expression of *Klf5 *(a member of the SP1 family) and *Hif1a*, *Smad3 *and *Smad4 *was simultaneously increased by *Stat3 *deletion, indicating the potential transcriptional complex formation among the corresponding transcription factors.

**Table 3:** Enriched TFBS In Genes Differentially Expressed in *Stat3*^Δ/Δ ^mice

*TFBS*	*Count (Diff_Gene)*	*Frequency (Diff_Gene)*	*Count (background)*	*Frequency (background)*	*P*-*Value*	Q-*Value*
V$STAT	863	0.97	786	0.79	4.93E-59	2.29E-56
V$EGRF	850	0.96	757	0.76	2.92E-57	1.35E-54
V$AHRR	706	0.80	544	0.54	4.04E-53	1.87E-50
V$SP1F	857	0.97	789	0.79	2.15E-49	9.96E-47
V$ZF5F	502	0.57	321	0.32	2.18E-48	1.01E-45
V$E2FF	850	0.96	778	0.78	1.30E-47	6.03E-45
V$HIFF	564	0.64	439	0.44	4.66E-30	2.16E-27
V$SREB	513	0.58	425	0.42	2.76E-17	1.28E-14
V$AP2F	492	0.56	420	0.42	4.93E-15	2.29E-12

Note: promoter sequence (-2000 – 0 bps) from 886 out of 1105 differentially genes were available for analysis. The P-Value is calculated using binomial distribution probability. 5 random sets of 1000 mouse gene -2kb promoter regions were used to calculate the background frequency. The Q-Value is calculated using single-step Bonferroni adjustment to control for the multiple comparison effect.

### Pathway analysis revealed known and novel functions of STAT3 in the lung

Pathway enrichment test is an unbiased way to answer the question, ''Are the differentially expressed genes enriched in certain pathways?'' To address this issue, we compared the overlap of differentially expressed genes in *Stat3*^Δ/Δ ^mice with the known biological pathways in KEGG (Kyoto Encyclopedia of Genes and Genomes) using 1) the mouse genome and 2) a list of genes shown to be least changed in response to the *Stat3 *deletion in the gene array as background. Results from both analyses were consistent. Jak-Stat Signaling Pathway, Apoptosis, Cytokine-Cytokine Receptor Interaction, Insulin Signaling Pathway, Mapk Signaling Pathway, Focal Adhesion, and Wnt Signaling Pathway were among the most enriched pathways identified from the analysis of differentially expressed genes (Table 4). mRNAs mediating steroid biosynthesis and fatty acid metabolism were mostly decreased in *Stat3*^Δ/Δ ^mice. The most overrepresented pathways and the differentially expressed genes associated with those pathways are illustrated in Additional files 2. The finding that known *Stat3 *functions, including the Jak-Stat Signaling Pathway were identified from microarray analysis of *Stat3*^Δ/Δ ^mice type II cells using knowledge integration approaches provides support for the utility of the analysis to detect novel pathways regulated by *Stat3*.

**Table 4:** Enriched Pathways In Genes Differentially Expressed in *Stat3*^Δ/Δ ^mice

*Term*	*Count*	*%*	*P-Value*
Jak-Stat Signaling Pathway	38	3.26	7.56E-09
Apoptosis	24	2.06	5.04E-06
Cytokine-Cytokine Receptor Interaction	43	3.69	2.59E-05
Insulin Signaling Pathway	28	2.40	5.53E-05
Mapk Signaling Pathway	45	3.86	8.51E-05
Focal Adhesion	37	3.17	1.55E-04
Wnt Signaling Pathway	28	2.40	1.68E-04

Overrepresented pathways were identified by comparison the overlap of differentially expressed genes and all genes in MOE430 mouse genome (reference) with the known KEGG pathways. A pathway is considered to be over-represented when a probability P-Value ≤ 0.01 and gene frequency (genes in the pathway/total number of differentially expressed genes) ≥ 2%.

### Stat3 influences protein metabolism in lung type II cells

''Protein metabolism'' was the most enriched biological process (5.83E-10), accounting for 20% of the induced genes caused by the deletion of *Stat3*. More than 40 genes encoding proteins involved in protein ubiquitination/ubiquitin cycle were present (Table 5). Of interest, Casitas B-lineage lymphoma b (*Cblb*) was increased 7.3 fold. CBLB is a member of Cbl ubiquitin ligases (E3) protein family that are tyrosine-phosphorylated in response to a wide variety of receptor mediated stimuli, including epidermal growth factor receptors, cytokine receptors such as colony stimulating factor family receptors (increased 2–3 fold in the *Stat3*^Δ/Δ ^cells) and immune complex receptors such as *Fcgr2b *(increased 2.7 fold). CBLB functions as an adaptor protein that regulates many signal-transduction pathways including JAK-STAT signaling pathways through its ability to interact with critical signaling molecules [22]. The expression of genes encoding known CBLB binding partners was significantly increased in type II cells from the *Stat3*^Δ/Δ ^mice, including SYK kinase (increased 4 fold), JAK2 (increased 2.1 fold), PKC (increased 2.6 fold), phosphatidylinositol 3-kinase (*Pik3r1 *increased 1.8 fold, *Pik3c2a *increased 3 fold), adenylate cyclase-associated protein 1 (*Cap1 *increased 5 fold) and *Crk *(increased 2.5 fold). These signaling molecules can in turn modulate various down stream signaling cascades including those mediated by small G-proteins (multiple members of RAS oncogene family were induced 2–3 fold), as well as PI3K/AKT mediated signaling and *Nfat *induction (increased 4.1 fold) [23-27]. CBLB physically interacts with another ubiquitin-protein ligase, NEDD4 (increased 3.8 fold), an interaction that reverses CBLB effects by targeting CBLB for ubiquitination and proteasomal degradation [28]. SLIM is a known E3 ubiquitin ligase specifically interacts with activated STAT1 and STAT4, inducing their ubiquitination and degradation [29]. We speculate that CBLB may act in a similar manner to regulate STAT3 signaling. In addition, SUMO1 and SUMO1 specific peptidase 2 were both increased more than 2 fold in *Stat3*^Δ/Δ ^mice. SUMO1 can covalently modify many targets including STAT1 and glucocorticoid receptor NR3C1 (increased 5 fold) and regulate protein stability and transcriptional activity [30]. Taken together, deletion of STAT3 activates a number of molecules regulating protein metabolism, stability and routing, indicating the potential role of ubiquitination and sumoylation in cytokine signaling and STAT3 activation that influences cellular adaptation.

**Table 5:** Genes In Ubiquitin Cycle Were Largely Induced In *Stat3*^Δ/Δ ^Mice

*UniGene ID*	*Fold*	*P-Value*	*Gene Symbol*	*Gene Title*
Mm.328206	7.29	9.98E-04	Cblb	Casitas B-lineage lymphoma b
Mm.260635	4.56	6.34E-03	Axot	Axotrophin
Mm.98668	3.79	6.93E-05	Nedd4l	neural precursor cell expressed, developmentally down-regulated gene 4-like
Mm.276229	3.37	1.41E-03	Fbxo30	F-box protein 30
Mm.258476	3.30	6.58E-03	Tbl1x	transducin (beta)-like 1 X-linked
Mm.28017	3.22	3.62E-03	Fbxw11	F-box and WD-40 domain protein 11
Mm.16974	3.03	1.66E-04	Usp47	ubiquitin specific peptidase 47
Mm.388965	3.01	8.38E-04	Rnf12	Ring finger protein 12
Mm.41711	2.87	1.43E-03	Pja2	Praja 2, RING-H2 motif containing (Pja2), transcript variant 2, mRNA
Mm.214746	2.75	1.78E-03	Fbxl3	F-box and leucine-rich repeat protein 3
Mm.242646	2.75	1.83E-03	Usp9x	ubiquitin specific protease 9, X chromosome
Mm.9002	2.70	6.26E-04	Ube3a	ubiquitin protein ligase E3A
Mm.30051	2.53	4.86E-04	Rnf10	Ring finger protein 10
Mm.362118	2.41	9.26E-04	Sumo1	SMT3 suppressor of mif two 3 homolog 1 (yeast) (Sumo1)
Mm.328135	2.36	3.35E-04	Rfwd2	Constitutive photomorphogenic protein (Cop1)
Mm.290908	2.34	3.27E-04	Birc6	baculoviral IAP repeat-containing 6
Mm.180052	2.34	4.84E-03	Ube2d2	Ubiquitin-conjugating enzyme E2D 2
Mm.391601	2.29	9.75E-03	Ube2d3	ubiquitin-conjugating enzyme E2D 3
Mm.4480	2.25	1.28E-03	Rbbp6	Retinoblastoma binding protein 6 (Rbbp6)
Mm.319512	2.24	5.65E-03	Hip2	huntingtin interacting protein 2
Mm.12665	2.19	1.60E-02	Cul3	cullin 3
Mm.244179	2.12	3.75E-03	Herc1	Hect (homologous to the E6-AP (UBE3A) carboxyl terminus) domain and RCC1 (CHC1)-like domain (RLD) 1
Mm.392272	2.10	4.55E-04	Smurf2	SMAD specific E3 ubiquitin protein ligase 2
Mm.297431	1.95	2.84E-03	Senp2	SUMO/sentrin specific peptidase 2
Mm.253542	1.94	1.53E-02	Rnf138	ring finger protein 138
Mm.235407	1.94	1.32E-02	Ube2v2	ubiquitin-conjugating enzyme E2 variant 2
Mm.392862	1.93	3.54E-03	Arih1	ariadne ubiquitin-conjugating enzyme E2 binding protein homolog
Mm.78312	1.89	1.07E-02	Wwp1	WW domain containing E3 ubiquitin protein ligase 1
Mm.44876	1.84	3.88E-03	Trim2	tripartite motif protein 2

### STAT3 influences phosphate metabolism and protein kinase cascade in lung type II cells

Expression of genes regulating ''Phosphate metabolism'' and ''protein kinase cascade'' were significantly increased in *Stat3*^Δ/Δ ^mice (3.1E-07 and 1.38E-06, respectively), accounting for 7.5% of total induced genes (Table 6). Expression of genes encoding a number of kinase that phosphorylate STAT3 in vivo or in vitro were increased [31-35], including Janus kinase 1 and 2 (increased 3.1 and 2.6 fold, respectively), ribosomal protein S6 kinase polypeptide 3 (*Rps6ka3*, increased 3.1 fold), met proto-oncogene (*Met*, increased 4.2 fold), mitogen activated protein kinase 8 (*Mapk8*, increased 4.7 fold) and Dual-specificity tyrosine-phosphorylation regulated kinase 1a (*Dyrk1a*, increased 2.6 fold). The increased expression of these genes indicates a potential compensatory mechanism related to the lack of activation of STAT3 or its targets.

**Table 6:** Genes In Phosphate Metabolism Were Largely Induced In *Stat3*^Δ/Δ ^Mice

*UniGene ID*	*Fold*	*P-Value*	*Gene Symbol*	*Gene Title*
Mm.188734	8.32	3.47E-05	Adk	Adenosine kinase
Mm.21495	4.70	1.82E-03	Mapk8	Mitogen activated protein kinase 8
Mm.3879	4.69	4.29E-05	Hif1a	Hypoxia inducible factor 1, alpha subunit
Mm.86844	4.18	1.89E-03	Met	Met proto-oncogene
Mm.225505	4.09	2.32E-03	Chka	Choline kinase alpha
Mm.375031	4.02	1.39E-03	Syk	Spleen tyrosine kinase
Mm.327591	3.60	1.26E-03	Cask	Calcium/calmodulin-dependent serine protein kinase
Mm.248647	3.42	2.36E-02	Pi4k2b	Phosphatidylinositol 4-kinase type 2 beta
Mm.255822	3.31	2.08E-04	Camk2d	Calcium/calmodulin-dependent protein kinase II, delta
Mm.25559	3.30	7.41E-03	Stk17b	Serine/threonine kinase 17b (apoptosis-inducing)
Mm.328476	3.15	1.45E-02	Rps6ka3	Ribosomal protein S6 kinase polypeptide 3
Mm.289657	3.14	1.66E-04	Jak1	Janus kinase 1
Mm.3810	3.08	7.64E-03	Pik3c2a	Phosphatidylinositol 3-kinase, C2 domain
Mm.202606	3.04	1.02E-04	Mast4	Microtubule associated serine/threonine kinase family member 4
Mm.309867	2.88	3.93E-04	Ptk9	Protein tyrosine kinase 9
Mm.280125	2.86	3.15E-03	Crk	v-crk sarcoma virus CT10 oncogene homolog (avian)
Mm.272548	2.82	4.37E-04	Etnk1	Ethanolamine kinase 1
Mm.197552	2.74	1.10E-03	Tgfbr1	Transforming growth factor, beta receptor I
Mm.262330	2.66	1.54E-02	Stk3	Serine/threonine kinase 3 (Ste20, yeast homolog)
Mm.295263	2.65	1.43E-03	Dcamkl1	Double cortin and calcium/calmodulin-dependent protein kinase-like 1
Mm.222178	2.57	2.58E-04	Prkca	Protein kinase C, alpha (Prkca), mRNA
Mm.310973	2.56	5.12E-04	Dyrk1a	Dual-specificity tyrosine-(Y)-phosphorylation regulated kinase 1a
Mm.313594	2.50	5.58E-03	Mpp5	Palmitoylated 5 (MAGUK p55 subfamily member 5)
Mm.6710	2.49	3.65E-02	Rock1	Rho-associated coiled-coil forming kinase 1
Mm.440470	2.46	3.16E-03	Crk7	CDC2-related kinase 7
Mm.389061	2.36	1.34E-03	Pftk1	PFTAIRE protein kinase 1 (Pftk1), mRNA
Mm.244236	2.34	8.50E-03	Pkn2	Protein kinase N2
Mm.288141	2.25	7.64E-03	Rp2h	Retinitis pigmentosa 2 homolog (human)
Mm.31672	2.21	4.65E-04	Cdk6	Cyclin-dependent kinase 6 (Cdk6), mRNA
Mm.215171	2.20	5.62E-04	Trpm6	Transient receptor potential cation channel, subfamily M, member 6
Mm.3994	2.18	3.98E-04	Dusp16	Dual specificity phosphatase 16
Mm.136511	2.17	5.10E-04	Tlk1	MKIAA0137 protein
Mm.332231	2.16	3.01E-03	Magi2	Membrane associated guanylate kinase, WW and PDZ domain containing 2
Mm.16340	2.13	1.50E-02	Fgfr2	Fibroblast growth factor receptor 2
Mm.275839	2.13	1.00E-02	Jak2	Janus kinase 2
Mm.393114	2.08	7.62E-04	Csnk1a1	Casein kinase 1, alpha 1
Mm.17918	2.08	7.94E-04	Marcksl1	MARCKS-like 1
Mm.368668	2.06	6.49E-04	Csnk1g3	Casein kinase 1, gamma 3 (Csnk1g3), mRNA
Mm.291936	2.01	8.02E-03	Map4k5	Mitogen-activated protein kinase kinase kinase kinase 5
Mm.35290	-2.00	1.42E-02	Ripk4	Receptor-interacting serine-threonine kinase 4
Mm.36006	-2.39	1.22E-03	Ak7	Adenylate kinase 7
Mm.306163	-2.68	8.63E-03	Prkar1b	Protein kinase, cAMP dependent regulatory, type I beta
Mm.7373	-2.71	1.05E-03	Per1	Period homolog 1 (Drosophila)
Mm.32831	-2.96	2.47E-02	Wif1	Wnt inhibitory factor 1
Mm.44442	-3.76	2.45E-04	Kndc1	Kinase non-catalytic C-lobe domain (KIND) containing 1

### STAT3 influences lipid homeostasis in lung type II cells

**Figure 1:**
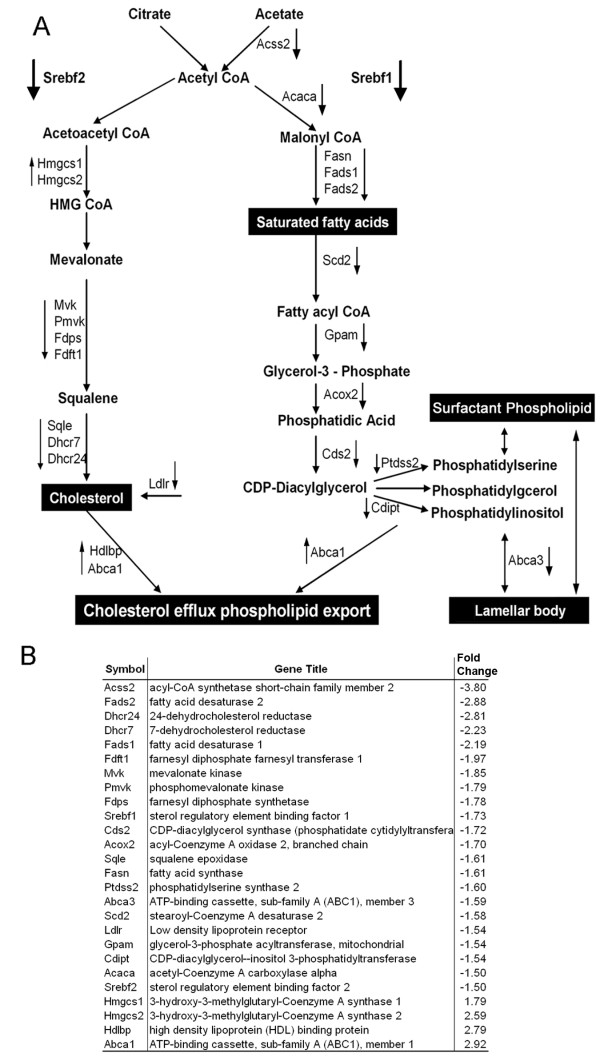
**Genes involved in lipids biosynthesis and clearance pathways were altered in type II cells from *Stat3*^Δ/Δ ^mice**. A. *Srebf1 *and *Srebf2 *regulate genes encoding the major metabolic enzymes in the fatty acid, cholesterol and phospholipids biosynthesis. The arrow (↑) indicates that the mRNA level was induced in *Stat3*^Δ/Δ ^mice; (↓) indicates the mRNA level was reduced in *Stat3*^Δ/Δ ^mice. Gene symbols, descriptions and the expression changes are listed in panel B.

Expression of genes encoding sterol regulatory element binding factor 1 and 2 (*Srebf1 *and *Srebf2*), their cleavage activating protein (*Scap*) and multiple SREBP target genes involved in lipid metabolism were decreased in *Stat3*^Δ/Δ ^mice type II cells. As depicted in Figure 1, genes dedicated to the biosynthesis of fatty acid, phospholipid and cholesterol were down regulated, with the exception of 3-hydroxy-3-methylglutaryl-Coenzyme A synthase (*Hmgcs*), which was increased. HMGCS participates in other metabolic pathways, including valine, leucine and isoleucine degradation (KEGG 00280). This analysis indicates that metabolic pathways regulating fatty acid, phospholipid, and cholesterol biosynthesis were coordinately decreased after deletion of *Stat3 *in type II cells, supporting an important role of STAT3 in regulating lipids biosynthesis in the lung. LDL receptor (*Ldlr*), mediating cholesterol uptake, and ATP-binding cassette A3 (*Abca3*), important for phospholipid transport, lamellar body formation and pulmonary surfactant secretion in alveolar type II cells were decreased in the *Stat3*^Δ/Δ ^mice cells [36-38]. Recent *in vitro *studies from our group confirmed the direct binding of SREBP1c to the Abca3 promoter (Besnard et.al, submitted for publication). On the other hand, the increased expression of *Abca1*, a key facilitator of cellular cholesterol and phospholipid export [39,40] and high density lipoprotein binding protein (*Hdlbp*), which may function in the removal of excess cellular cholesterol, suggests that cholesterol and phospholipid clearance were induced after deletion of *Stat3*. Adenosine kinase (*Adk*), was increased 8.2 fold. Cellular role of ADK in lipid metabolism is somewhat controversial [41], *Adk *deficient mice developed neonatal hepatic steatosis and die within 14 days with fatty liver [42]. Increased expression of lipid export genes and decreased expression of genes mediating lipid biosynthesis likely cause a reduction of total lipids level in the type II cells of *Stat3*^Δ/Δ ^mice.

**Figure 2:**
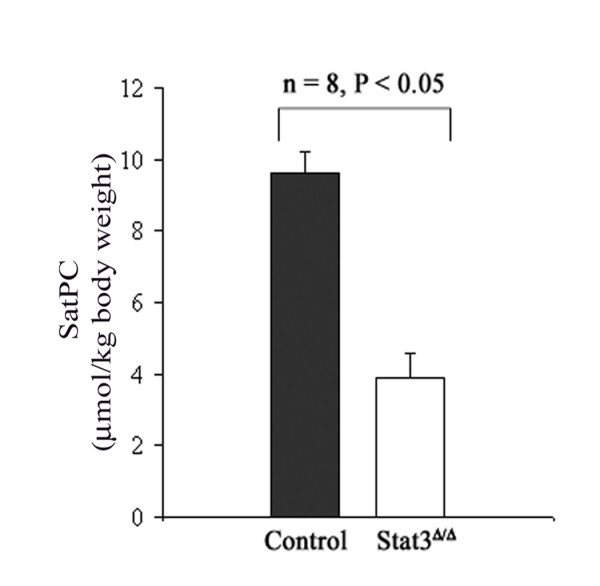
**Surfactant saturated phosphatidylcholine (SatPC)** was significantly decreased in bronchoalveolar lavage fluid from the *Stat3*^Δ/Δ ^mice, n = 8 per group. Aliquots of BALF were extracted with chloroform/methanol (2:1) and SatPC was isolated with osmium tetroxide followed by measurement of phosphorus as described previously [12]. Statistical differences were analyzed by Student t-test.

Previous studies demonstrated the susceptibility of *Stat3*^Δ/Δ ^mice to lung injury and death related to surfactant dysfunction [12]. Consistent with our prediction from the present mRNA microarray analysis, the saturated phosphatidycholine (SatPC) content in bronchoalveolar lavage fluid was significantly decreased in *Stat3*^Δ/Δ ^mice (Figure 2). Significantly decreased SatPC synthesis and abnormalities in lamellar body numbers and morphology were also observed in *Stat3*^Δ/Δ ^mice (data not shown). Taken together, a number of genes regulating surfactant lipid homeostasis were altered in type II cells isolated from the Stat3^Δ/Δ ^mice, consistent with biochemical, functional, and morphologic changes in the surfactant system that is exacerbated by oxidant stress [12] or expose to pathogens [13].

### Role of Akt in Stat3 regulated lipid metabolism

Our observations support the view that the decrease in SREBP, at least in part, results in decreased expression of genes regulating lipid biosynthesis and metabolism in type II cells from *Stat3*^Δ/Δ ^mice. SREBPs are master regulators of lipid metabolism. The transcriptional targets, and the pathways mediated by SREBP in liver have been well studied [43]. SREBPs are expressed in the developing lung, SREB1c increases in the developing lung concomitantly with the perinatal increase in surfactant and lipid synthesis, surfactant protein and *Abca3 *expression, genes critical for surfactant function.

However, the role of Stat3 in regulating SREBP and associated lipid metabolism in the lung is largely unknown. In the present analysis, we sought to identify mechanisms by which *Stat3 *regulates SREBPs and associated lipid biosynthesis pathways in alveolar type II cells in the lung. The regulation of SREBPs occurs at both transcriptional and post-transcriptional levels. The post-transcriptional regulation requires SCAP. Cre-mediated disruption of *Scap *significantly reduced *Srebf1 and 2 *levels as well as SREBP target gene expression in liver [44]. There are multiple potential STAT sites on the *Scap *promoter. Thus, *Stat3 *may influence lipid biosynthesis through the transcriptional regulation of *Scap*. At transcriptional level, nuclear hormone receptors (*Nr1h2 and Nr1h3*) and Pgc1-alpha and beta are known to regulate SREBPs expression in liver. Increased expression of *Srebf1 *and *Fasn *was associated with increased hepatic triglyceride content in *Stat3 *deficient mice [10,45]. In the present study, expression of SREBP and their down stream targets were decreased in type II cells from *Stat3*^Δ/Δ ^mice (a finding that contrast with the findings in the liver) without changes in other known regulators such as *Nr1h2, Nr1h3*, *Pgc1-alpha *or *Pgc1-beta*, indicating the likely presence of alternative regulatory mechanisms in lung cells.

We hypothesize that AKT plays an important role in *Stat3 *regulated SREBP expression and associated lipogenesis in lung based on the following observations: 1) Protein and mRNA levels of *Akt *were increased in cells constitutively expressing active *Stat3 *and were reduced after *Stat3 *depletion [46]. STAT3 binds to *Akt1 *promoter was confirmed by ChIP assay [47], suggesting that A *kt *maybe a direct transcriptional target of *Stat3*. AKT, on the other hand, inhibits *Stat3 *transcriptional activity and phosphorylation [48]. Decreased *Akt *gene expression seen after deletion of *Stat3 *may represent a direct effect of *Stat3 *deficiency or to a negative regulatory response to STAT3 deficiency, 2) AKT activation induces both *Srebf1 *and *Srebf2 *mRNAs and proteins as well as key enzymes in the cholesterol, fatty acid and membrane lipid biosynthesis pathways [49], 3) Multiple lines of evidence suggest that PI3K influences *Stat3 *activation. STAT3 binds directly to the PI3K regulatory subunits [50-52]. The expression of genes encoding for several PI3K subunits were altered after deletion of *Stat3 *(see Additional file 1), supporting the involvement of PI3K/Akt signaling in *Stat3 *regulated bioprocesses in lung, and 4) Since AKT physically interacts with FOXA2 and regulates FOXA2-dependent transcriptional activity [53], the effects of AKT may be mediated, in part, via *Foxa2. Foxa2 *expression was reduced more than 2 fold in cells from the *Stat3*^Δ/Δ ^mice. FOXA2 regulates lipid metabolism in both lung and liver [54,55]. Deletion of either *Stat3 *or *Foxa2 *resulting in decreased expression of a number of the overlapping genes that play important roles in surfactant homeostasis including Abca3 [55]. Thus, interactions between AKT and FOXA2 represent another potential mechanism by which lipid metabolism is influenced in *Stat3*^Δ/Δ ^mice.

### Stat3 influences expression of genes mediating apoptosis and cell survival

**Figure 3:**
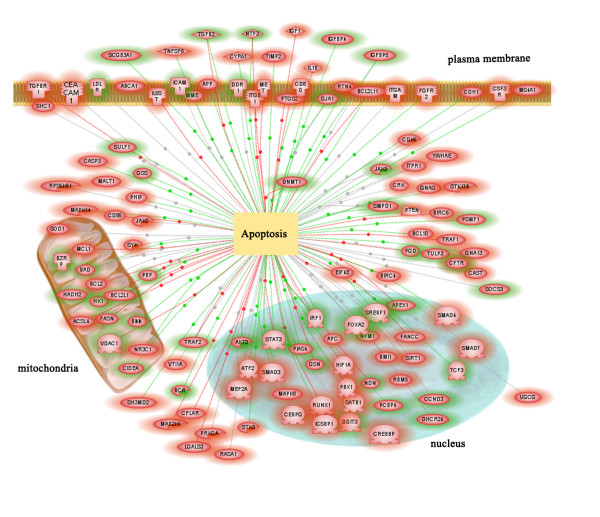
**Genes involved in apoptotic pathways were altered in type II cells isolated from *Stat3*^Δ/Δ ^mice**. Genes associated with apoptosis were identified via literature mining using Pathway Studio 4.0 (Ariadne Genomics, Inc.). STAT3 regulates apoptosis positively and negatively, via processes occurring in multiple intracellular compartments. Genes up-regulated in *Stat3*^Δ/Δ ^mice are framed in red. Genes down-regulated in *Stat3*^Δ/Δ ^mice are framed in green. Each dashed line demarcated with a larger dot linking "apoptosis" and each gene node indicates a regulatory relationship based on the literature references. Regulatory relationships are denoted by line colors (green, positive  regulation; red, negative regulation; and gray, regulation direction is  unknown).

Many genes modulating apoptosis/cell survival were altered in response to the deletion of *Stat3 *from type II cells, including multiple Bcl-2 family members (*Bcl2, Bcl2l1, Mcl1, Bcl2l11 *and *Bad*), caspase 3 (*Casp3*), FADD-like apoptosis regulator (*Cflar*, also known as Flip), mucosa associated lymphoid tissue lymphoma translocation gene 1 (*Malt1*), prostaglandin-endoperoxide synthase 2 (*Ptgs2*) and nuclear receptor subfamily 3, group C, member 1 (glucocorticoid receptor, *Nr3c1*). In depth literature mining identified more apoptosis related genes than did analysis by Gene Ontology annotation. As depicted in Figure 3, our study indicates that STAT3 regulates apoptosis in a complex manner via processes that occur in multiple intracellular locations. Thus, STAT3 appears to serve as key regulator of apoptosis in alveolar type II cells. Among those apoptosis related genes, *Malt1, Ptgs2 *and *Nr3c1 *were strongly induced (> 5 fold in compare with control). MALT1 interacts with BCL10 (increased 2 fold in *Stat3*^Δ/Δ ^mice). The formation of this complex is essential for NF-kappaB activation that, in turn, play a role in cell survival. IKKB phosphorylates BCL10 in its MALT1 interaction domain, causing BCL10 and MALT1 to disassociate, resulting in attenuation of NFKB signaling and cytokine production [56,57]. PTGS2 also known as COX-2, a key enzyme in prostaglandin biosynthesis, that is highly expressed in alveolar type II cells. The expression of *Ptgs2 *is increased in epithelial tumors, including non-small cell lung and prostate cancers via activation of the IL-6/GP130/*STAT3 *signaling pathway [58]. This pathway could contribute to tumor formation by promotion of tumor cell resistance to apoptosis via inhibitor of apoptosis (IAP)-dependent mechanism [58,59]. Consistent with these observations, *Ptgs2*, *Il6st *and two of the IAP family members (*Birc4 *and *Birc6*) were correspondingly induced in *Stat3*^Δ/Δ ^cells (5.7, 1.7, 3.2 and 2.3 fold respectively). *Nr3c1 *(increased 5.1 fold) encodes a receptor for glucocorticoids that can act as both a transcription factor and as a regulator of other transcription factors. STAT3 and NR3C1 physically interact to mediate effect of glucocorticoid on the IL-6-mediated inflammatory response [60-62]. NR3C1 also interacts with stress-responsive transcription factors (*Hif1a*, increased 4.7 fold), mitogen activated protein kinase 8 (*Mapk8 or Jnk*, increased 4.7 fold) and tyrosine 3-monooxygenase/tryptophan 5-monooxygenase activation protein, epsilon polypeptide (*Ywhal*, increased 3.3 fold), a 14-3-3 family of proteins implicated in the pathogenesis of small cell lung cancer [63,64].

STAT3 is likely to regulate apoptosis by multiple mechanisms including gene transcription. Bcl-xL is the direct transcription target of STAT3[65]. STAT3 can serve as an anti-apoptotic factor by transcriptional up-regulating the expression of Bcl-xL [66]. The decrease of Bcl-xL may represent a direct response to *Stat3 *deletion. The fact that expression of Bcl-xL blocked the apoptotic effects of the adenovirus in lung injury suggested that Bcl-xL may mediate the role of STAT3 in the regulation and survival of the respiratory epithelium [13]. The Pi3k-Akt pathway represents a second mechanism by which STAT3 influences cytoprotection. Pi3k-Akt signaling mediates a wide range of down stream targets to regulate apoptosis [67]. For example, AKT phosphorylates multiple Bcl-2 family members, including BAD and Bcl-xL [68], inhibits caspase 3 activation [66] and blocks cytochrome C release from mitochondria [69]. Another mechanism by which STAT3 modulates apoptosis is through protein-protein interactions. Bcl-xL is a direct transcriptional target of STAT3; Bcl-xL interacts with VDAC1 to regulate the outer mitochondrial membrane channel induce apoptosis [70,71]. CASP3 can interact with multiple apoptosis proteins including CFLAR, BIRC4 and 6, BCL2 and APP [72-75]. The expression of *Casp3 *as well as its interaction partners was induced in *Stat3*^Δ/Δ ^mice. NR3C1 has both pro- and anti-apoptotic effects. NR3C1 physically interact with STAT3, HIF1A, MAPK8, YWHAL [63,64,76]; these stress-responsive transcription factors and signaling molecules were largely induced in the present array from Stat3^Δ/Δ ^type II alveolar epithelial cells. The close transcriptional communication and physical interactions among these transcriptional regulators likely play a critical role in regulating the balance of apoptosis and cell survival. In the present study, effects of STAT3 deletion were assessed in type II epithelial cells purified from the adult mouse lung. mRNA was isolated immediately after isolation to avoid cell culture dependent alteration in gene expression. It is possible that the cells have undergone added cellular stress during protease treatment, isolation and purification, which in turn may influence the expression of genes. Our results support the view that STAT3 regulates the balance between a subset of pro- and anti-apoptotic genes, determining the cell death or survival through multiple mechanisms. Consistent with the present microarray prediction, cleaved caspase-3 and TUNEL positive cells were significantly increased in Stat3^Δ/Δ ^mice following adenoviral infection and the apoptosis can be blocked by expression of Bcl-xL [13].

## Conclusion

**Figure 4:**
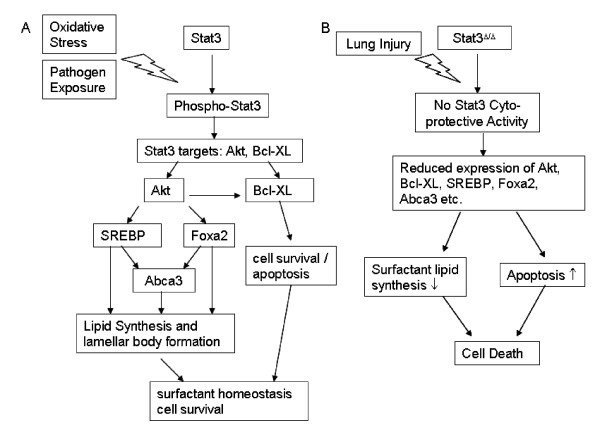
**Schematic representation of the proposed role of Stat3 in cytoprotection of the lung in normal (A) versus *Stat3*^Δ/Δ ^mice (B)**. Stat3 plays critical role in cyto-protection during lung injury [12, 13]. Present data support the role of Stat3 in enhancing epithelial cell survival and surfactant lipid synthesis that contribute to the maintenance of lung function. For simplicity, only representative genes were included.

Our previous studies demonstrated that Stat3 plays critical role in cyto-protection during lung injury [12,13]. Present data support the role of Stat3 in enhancing epithelial cell survival and surfactant lipid synthesis that contribute to the maintenance of lung function. Deletion of *Stat3 *from type II alveolar epithelial cells induced the expression of the genes regulating protein metabolism, protein transport, chemotaxis and apoptosis while decreasing the expression of genes regulating lipid synthesis and metabolism. Critical to pulmonary function during injury, Stat3 influences the expression of genes regulating surfactant lipid synthesis and surfactant homeostasis including Abca3. As illustrated in Figure 4, the present study identified a complex regulatory network by which *Stat3 *regulates gene expression in type II alveolar cells that is required for cellular homeostasis following injury. STAT3 likely interacts with AKT/FOXA2 in the regulation a number of biological processes in alveolar type II cells, including cell survival/apoptosis, cholesterol and fatty acid biosynthesis required for surfactant homeostasis and lung function.

## Methods

### Transgenic Mice

*SP-C-rtTA/(tetO)7CMV-Cre*/*Stat3*^flx/flx ^triple-transgenic mice were generated as described previously [12]. *Stat3*flx/flx mice were a kind gift of Dr. Takeda (Hyogo College of Medicine, Japan) [8]. In the presence of doxycycline, exon 21 of the *Stat3 *gene is permanently deleted from respiratory epithelial cells prior to birth (*Stat3*^Δ/Δ ^mice) [12]. *Stat3 *deleted transgenic (*Stat3*^Δ/Δ^) and non-deleted littermates (double transgenic,*SP-C-rtTA, or tetO7CMV-Cre *and *Stat3*^flx/flx^) were used for the experiments. Doxycycline was administered to the dams in the food at a concentration of 625 mg/kg (Harlan Teklad, Madison, WI) from embryonic day 0 (E0) to postnatal day 25 (P25), resulting in extensive deletion of *Stat3 *in respiratory epithelial cells [12]. As previously described, deletion of *Stat3 *did not alter lung size, morphology or survival under non-stressed condition [13].

### RNA Extraction

Alveolar type II cells were isolated from 8 weeks old, sex and age matched littermate control and *Stat3*^Δ/Δ ^mice using collagenase and differential plating as described by Rice *et al.*[77]. Type II cells from 3 mice were pooled to obtain one cell pellet. Three independent pools were generated from control and *Stat3*^Δ/Δ ^mice separately for purification of RNA and microarray hybridization. Type II cells were homogenized with TRIzol reagent (Invitrogen, Carlsbad, CA). RNA concentration was measured by spectrophotometer and normalized prior to cDNA synthesis. These cell isolates consist of more than 90% alveolar type II cells with residual alveolar macrophages as the major contaminating cell. Purity was assessed by modified Papanicolaou stain. Purity and number of type II cells isolated from *Stat3*^Δ/Δ ^mice were not different from controls.

### RNA Microarray Analysis

mRNA was extracted from three independent pools of isolated type II cells from adult *Stat3*^Δ/Δ ^and control mice. The cRNA was then hybridized to the murine genome MOE430 (consists of ≈ 45000 gene entries) chips (Affymetrix, Santa Clara, CA) according to the manufacturer's protocol. The RNA quality and quantity assessment, probe preparation, labeling, hybridization and image scan were carried out in the CCHMC Affymetrix Core using standard procedure. RNA quality and quantity were analyzed by spectrophotometer. The A260/A280 ratio was used to determine RNA purity with the acceptable region of 1.9–2.1. Affymetrix Microarray Suite 5.0 was used to scan and quantitate the gene chips under default scan settings. Normalization was performed using the Robust Multichip Average model [78,79]. Data were further analyzed using affylmGUI from R/Bioconductor package [80]. Differentially expressed genes were selected with the threshold of T-Test P-value ≤ 0.05, False Discovery Rate (FDR) ≤ 10% and fold change ≥ 1.5. We prioritized the mRNAs whose abundance consistently changed in multiple probe sets by selecting them without the FDR consideration. Unknown cDNA clones/ESTs and duplicated gene entries were removed from further functional analysis.

### Gene Ontology Analysis

Gene Ontology Analysis was performed using public available web-based tool David (database for annotation, visualization, and integrated discovery) [81]. Overrepresented biological processes were selected at the threshold of Fisher Exact Test P-Value ≤ 0.05 and minimum gene counts belonging to an annotation term ≥ 2%.

### Promoter Analysis: Transcription Factor Binding Sites Search

Promoter sequences of all differentially expressed genes (-2000 bp upstream from the tentative TSS) were retrieved from our database (originally downloaded from UCSC Genome browser) and searched for over-represented TFBS (Transcription Factor Binding Site) in these sequences using MatInspector (Genomatix) using the complete Vertebrate Matrix Library 6.2. The P value is calculated using binomial distribution probability by comparing the matrix match of the promoter regions of differentially expressed genes with the promoters from random mouse genes sets (-2kb for all the promoters). The single-step Bonferroni adjustment is used to control for the multiple comparison effect (i.e., multiplication of p-value by the number of TFBS in Genomatix Vertebrate Matrix Library).

### Pathway Enrichment Test

Overrepresented pathways were identified by comparison the overlap of differentially expressed genes and all genes in MOE430 mouse genome (reference) with the known KEGG pathways. A Fisher's exact test for 2·2 contingency table was used to calculate the statistical significance. A pathway is considered to be over-represented when a probability P value = 0.01 and gene frequency (genes in the pathway/total number of differentially expressed genes) ≥ 2%.

### Literature Mining

Potential protein-protein or protein-DNA interactions were identified using Pathway Studio (Ariadne Genomics) that contains MedScan, an automated text-mining tool to search the entire PubMed and other public sources (The current version database contains more than 15 million Medline abstracts/full text). Gene expression profiling results was imported into the Studio and used to interpret pathways, gene regulation networks, and protein interaction maps.

### Validation of mRNAs

Real-time RT-PCR was used to cross validate changes in a subset of genes from microarray selection. mRNA was extracted from alveolar type II epithelial cells isolated from *Stat3*^Δ/Δ ^and control mice using RNeasy Protect mini kit (Qiagen, Valencia, CA) according to the manufacturer's protocol. RNA concentration was measured by spectrophotometer. cDNA was made with SuperScript First-Strand Synthesis System (Invitrogen, Carlsbad, CA). *Malt1, Rtn4, Reg3g, Bcl2l1, Abca3, Scap, Fasn, Srebf1 and Srebf2 *were detected using primers listed Changes in mRNA were determined in type II cells isolated from *Stat3*^Δ/Δ ^and controls (*n *= 3–4/group). The following primers were used: *Malt1*: forward, TAT CCA GGA GGA CCC CAT GT and reverse, TCT GAT CAA AGC CAG TTA GCA TCAT; Rtn4: forward, AAG TGG AAG GAG TTT GAG AGA GCA and reverse, CTG TCT CAA AGC AGA TGT GAA AGC; *Reg3g*: forward, TGC CAA AAG AGC CCT CAG GA and reverse, TGC CTG AGG AAG AGG AAG GAT TCG; *Bcl2l1*: forward, TCT CTC TCC TCT GTC CAC CCT TG and reverse, TGC CCC TCA GAA GCC AGA AC; *Abca3*: forward, GCA TTG CCC TCA TTG GAG AGC CTG and reverse, TCC GGC CAT CCT CAG TGG TGG G; *Scap*: forward, TGA CCA CAA ACA AGG AGA GC and reverse, CAG GAA CAC CAA ACA GCA AG; *Srebf1*: forward, AAG CCG GGT GGG CGC CGG CGC CAT and reverse, GTC GTT CAA AAC CGC TGT GTC CAG; *Srebf2*: forward, CAT CCA GCA GCC TTT GAT ATA CCA G and reverse, AGG ACC GGG ACC TGC TGC ACC TGT G. *Fasn*: forward, GGA CAT GGT CAC AGA CGA TGA C and reverse, GTC GAA CTT GGA CAG ATC CTT CA. The PCR product was separated by the 1% agarose gel electrophoresis. β-actin was used as the internal control. Taqman Gene Expression Assay were used to confirm the expression of *Akt2, Cdipt, Acox2, Cds2 and Gpam *using Applied Biosystems 7300 Real-Time PCR System and company designed probes (Applied Biosystems). Statistical differences were determined using unpaired Student's t-tests.

## Supplementary Material

Additional file 1 **Genes Differentially Expressed in *Stat3*^Δ/Δ ^Mice**. Additional file descriptions text (including details of how to view the file, if it is in a non-standard format). Click here for file

Additional file 2 **Differentially Expressed Genes In Jak-Stat, Insulin and Wnt Signaling Pathway**. Pathways were downloaded from KEGG website [82]. Each rectangle represents one or multiple gene products. Genes up-regulated in *Stat3*^Δ/Δ ^mice are highlighted in red. Genes down-regulated in *Stat3*^Δ/Δ ^mice are highlighted in blue. Rectangle contains more than one gene products and changes expression in opposite directions are highlighted in yellow. Click here for file
