# Supplementary material for: Gene expression and biological processes influenced by deletion of Stat3 in pulmonary type II epithelial cells
Source: BMC Genomics. 2007 Dec 10;8:455. doi: 10.1186/1471-2164-8-455 (PMC2234434; doi:10.1186/1471-2164-8-455)
Supplement: Additional file 1 — Genes Differentially Expressed in Stat3Δ/Δ Mice. Additional file descriptions text (including details of how to view the file, if it is in a non-standard format). [file 1471-2164-8-455-S1.doc]

| Supplementary Table 1. Genes Differentially Expressed in *Stat3*^Δ/Δ^ Mice | | | |  |
| --- | --- | --- | --- | --- |
| *Gene Symbol* | *RefSeq* | *Gene Title* | *Fold* | *Pvalue* |
| Reg3g* | NM_011260 | regenerating islet-derived 3 gamma | -6.02 | 0.00019511 |
| 1700112E06Rik | XM_484353 | RIKEN cDNA 1700112E06 gene | -4.83 | 0.0000237 |
| 1110020P15Rik | NM_197979 | RIKEN cDNA 1110020P15 gene | -4.19 | 0.00032556 |
| Fmod | NM_021355 | fibromodulin | -4.13 | 0.0004796 |
| Upk3b | NM_175309 | uroplakin 3B | -3.98 | 0.00541479 |
| Acss2 | NM_019811 | acyl-CoA synthetase short-chain family member 2 | -3.80 | 0.036677677 |
| 2410012C07Rik | NM_177261 | RIKEN cDNA 2410012C07 gene | -3.76 | 0.00024475 |
| U46068 | NM_001012392 | cDNA sequence U46068 | -3.69 | 0.00253426 |
| 6430537H07Rik | NM_178689 | RIKEN cDNA 6430537H07 gene | -3.59 | 0.00156584 |
| Sox2 | NM_011443 | SRY-box containing gene 2 | -3.52 | 0.00118927 |
| Acas2 | NM_019811 | acetyl-Coenzyme A synthetase 2 (ADP forming) | -3.45 | 0.01886932 |
| BC038167 | XM_196478 | cDNA sequence BC038167 | -3.44 | 0.00015721 |
| BC004853 | NM_146117 | cDNA sequence BC004853 | -3.37 | 0.0004474 |
| Cldn4 | NM_009903 | claudin 4 | -3.32 | 0.00019161 |
| Gm1060 | XM_355721 | gene model 1060, (NCBI) | -3.32 | 0.00044789 |
| Gm107 | XM_129770 | gene model 107, (NCBI) | -3.32 | 0.0007664 |
| 4732473B16Rik | NM_175307 | RIKEN cDNA 4732473B16 gene | -3.25 | 0.0001632 |
| Scgb3a1 | NM_054037 | secretoglobin, family 3A, member 1 | -3.24 | 0.00215668 |
| C2 | NM_013484 | Complement component 2 (within H-2S), mRNA | -3.14 | 0.00083789 |
| Dnali1 | NM_175223 | dynein, axonemal, light intermediate polypeptide 1 | -3.07 | 0.00179649 |
| Dnahc6 | XM_287612 | dynein, axonemal, heavy chain 6 | -3.03 | 0.00131217 |
| Lrrc50 | NM_026648 | leucine rich repeat containing 50 | -3.03 | 0.00082316 |
| 3100002J23Rik | --- | RIKEN cDNA 3100002J23 gene | -3.01 | 0.00119244 |
| Gm872 | XM_354560 | gene model 872, (NCBI) | -3.00 | 0.0007989 |
| Arhgdig | NM_008113 | Rho GDP dissociation inhibitor (GDI) gamma | -2.97 | 0.00012973 |
| Wif1 | NM_011915 | Wnt inhibitory factor 1 | -2.96 | 0.02465999 |
| Igfbp5 | NM_010518 | insulin-like growth factor binding protein 5 | -2.95 | 0.00528194 |
| Foxj1 | NM_008240 | forkhead box J1 | -2.92 | 0.00227797 |
| 6820408C15Rik | NM_177656 | RIKEN cDNA 6820408C15 gene | -2.89 | 0.00055406 |
| Fads2 | NM_019699 | fatty acid desaturase 2 | -2.88 | 0.00162776 |
| Fbln1 | NM_010180 | fibulin 1 | -2.88 | 0.00069081 |
| 1700021K14Rik | NM_013785 | RIKEN cDNA 1700021K14 gene | -2.88 | 0.00718679 |
| Col1a2 | NM_007743 | procollagen, type I, alpha 2 | -2.88 | 0.0030736 |
| Cyp2s1 | NM_028775 | cytochrome P450, family 2, subfamily s, polypeptide 1 | -2.87 | 0.00347009 |
| Plunc | NM_011126 | palate, lung, and nasal epithelium carcinoma associated | -2.87 | 0.00348642 |
| Cyp2a4 | NM_007812 | cytochrome P450, family 2, subfamily a, polypeptide 4 | -2.85 | 0.00052562 |
| Muc5b | NM_028801 | mucin 5, subtype B, tracheobronchial | -2.84 | 0.00048346 |
| 1110020C03Rik | NM_026789 | RIKEN cDNA 1110020C03 gene | -2.83 | 0.00071812 |
| 2310076L09Rik | NM_025874 | RIKEN cDNA 2310076L09 gene | -2.83 | 0.0002536 |
| Chad | NM_007689 | chondroadherin | -2.83 | 0.00050001 |
| Spata5l1 | NM_001033256 | spermatogenesis associated 5-like 1 | -2.83 | 0.00019075 |
| Rbp1 | NM_011254 | retinol binding protein 1, cellular | -2.82 | 0.00128368 |
| 4933405I11Rik | NM_080457 | RIKEN cDNA 4933405I11 gene | -2.81 | 0.00656151 |
| Dhcr24 | NM_053272 | 24-dehydrocholesterol reductase | -2.81 | 0.00079047 |
| Aacs | NM_030210 | acetoacetyl-CoA synthetase | -2.77 | 0.00012958 |
| Cp | NM_007752 | ceruloplasmin | -2.76 | 0.00089671 |
| 1700010L19Rik | XM_128462 | RIKEN cDNA 1700010L19 gene | -2.72 | 0.00043764 |
| Per1 | NM_011065 | period homolog 1 (Drosophila) | -2.71 | 0.00104575 |
| Gdpd2 | NM_023608 | glycerophosphodiester phosphodiesterase domain containing 2 | -2.70 | 0.00105855 |
| Ccdc19 | XM_355284 | coiled-coil domain containing 19 | -2.70 | 0.00124955 |
| Prkar1b | NM_008923 | protein kinase, cAMP dependent regulatory, type I beta | -2.68 | 0.00862719 |
| Steap1 | NM_027399 | six transmembrane epithelial antigen of the prostate 1 | -2.68 | 0.0003103 |
| Steap4 | NM_054098 | STEAP family member 4 | -2.67 | 0.00137219 |
| Gna14 | NM_008137 | guanine nucleotide binding protein, alpha 14 | -2.65 | 0.00095568 |
| 1700016K19Rik | NM_198637 | RIKEN cDNA 1700016K19 gene | -2.65 | 0.00083325 |
| Lipg | NM_010720 | lipase, endothelial | -2.65 | 0.00103238 |
| 1810010G06Rik | XM_486167 | RIKEN cDNA 1810010G06 gene | -2.64 | 0.00030019 |
| Scnn1b | NM_011325 | sodium channel, nonvoltage-gated 1 beta | -2.64 | 0.0018774 |
| Cyp2b10 | NM_009998 | cytochrome P450, family 2, subfamily b, polypeptide 10 | -2.62 | 0.00350699 |
| Dnahc1 | --- | dynein, axonemal, heavy chain 1 | -2.62 | 0.00079034 |
| Calml4 | NM_138304 | calmodulin-like 4 | -2.60 | 0.00138018 |
| Rarres2 | NM_027852 | retinoic acid receptor responder (tazarotene induced) 2 | -2.58 | 0.02112368 |
| 2310007B03Rik | NM_172411 | RIKEN cDNA 2310007B03 gene | -2.57 | 0.00103261 |
| Ctxn1 | NM_183315 | cortexin 1 | -2.56 | 0.00137203 |
| BC022765 | NM_146026 | cDNA sequence BC022765 | -2.56 | 0.00102078 |
| Wdr40b | NM_178739 | WD repeat domain 40B | -2.55 | 0.00088125 |
| 4933404O19Rik | XM_126529 | RIKEN cDNA 4933404O19 gene | -2.53 | 0.00052456 |
| Gabrp | NM_146017 | gamma-aminobutyric acid (GABA-A) receptor, pi | -2.53 | 0.00076483 |
| Cidea | NM_007702 | cell death-inducing DNA fragmentation factor, alpha subunit-like effector A | -2.52 | 0.00086741 |
| BC024561 | NM_153576 | cDNA sequence BC024561 | -2.51 | 0.00039692 |
| Armc3 | XM_130012 | armadillo repeat containing 3 | -2.51 | 0.00231985 |
| 1700019F09Rik | NM_027963 | RIKEN cDNA 1700019F09 gene | -2.49 | 0.00169671 |
| Fndc1 | XM_354975 | fibronectin type III domain containing 1 | -2.47 | 0.00048076 |
| Ndn | NM_010882 | necdin | -2.47 | 0.00077561 |
| Osmr | NM_011019 | oncostatin M receptor | -2.46 | 0.00242896 |
| 1700026L06Rik | NM_027283 | RIKEN cDNA 1700026L06 gene | -2.44 | 0.00137757 |
| 9230110J10 | --- | hypothetical protein 9230110J10 | -2.43 | 0.00022208 |
| 4930526H21Rik | XM_205565 | RIKEN cDNA 4930526H21 gene | -2.42 | 0.00135461 |
| Gsta2 | NM_008182 | glutathione S-transferase, alpha 2 (Yc2) | -2.42 | 0.00555219 |
| Cpe | NM_013494 | carboxypeptidase E | -2.42 | 0.00056706 |
| Freq | NM_019681 | frequenin homolog (Drosophila) | -2.42 | 0.00050356 |
| 0610009K11Rik | NM_026689 | RIKEN cDNA 0610009K11 gene | -2.41 | 0.00040816 |
| BC038479 | NM_153803 | cDNA sequence BC038479 | -2.40 | 0.00067805 |
| Bace2 | NM_019517 | beta-site APP-cleaving enzyme 2 | -2.39 | 0.00044051 |
| Ak7 | XM_911194 | adenylate kinase 7 | -2.39 | 0.00122482 |
| C1s | NM_144938 | complement component 1, s subcomponent | -2.38 | 0.0005748 |
| Ttc18 | XM_127606 | tetratricopeptide repeat domain 18 | -2.37 | 0.00035683 |
| Tulp3 | NM_011657 | tubby-like protein 3 | -2.37 | 0.00039761 |
| Pcolce | NM_008788 | procollagen C-proteinase enhancer protein | -2.36 | 0.00992867 |
| Lrpb7 | NM_013588 | leucine rich protein, B7 gene | -2.36 | 0.0012979 |
| Prom1 | NM_008935 | prominin 1 | -2.36 | 0.0019177 |
| 2010004A03Rik | NM_029646 | RIKEN cDNA 2010004A03 gene | -2.35 | 0.0010155 |
| 1600029I14Rik | XM_135038 | RIKEN cDNA 1600029I14 gene | -2.32 | 0.00043501 |
| 2010001J22Rik | NM_001013022 | RIKEN cDNA 2010001J22 gene | -2.32 | 0.00043226 |
| Gtf2h4 | NM_010364 | general transcription factor II H, polypeptide 4 | -2.31 | 0.01586111 |
| Cyp4f15 | NM_134127 | cytochrome P450, family 4, subfamily f, polypeptide 15 | -2.31 | 0.00291011 |
| Cyp2b10 | NM_009998 | cytochrome P450, family 2, subfamily b, polypeptide 10 | -2.31 | 0.00723638 |
| Asns | NM_012055 | asparagine synthetase | -2.30 | 0.00057021 |
| Dnaic1 | NM_175138 | dynein, axonemal, intermediate chain 1 | -2.29 | 0.00121881 |
| Sulf1 | NM_172294 | sulfatase 1 | -2.29 | 0.00936152 |
| 1810049O03Rik | NM_028175 | RIKEN cDNA 1810049O03 gene | -2.27 | 0.00412903 |
| Igfbp4 | NM_010517 | insulin-like growth factor binding protein 4 | -2.27 | 0.00922662 |
| Prss11 | NM_019564 | protease, serine, 11 (Igf binding) | -2.26 | 0.0037573 |
| Calml3 | NM_027416 | calmodulin-like 3 | -2.26 | 0.02562625 |
| Fbn1 | NM_007993 | fibrillin 1 | -2.25 | 0.02512671 |
| Dhcr7 | NM_007856 | 7-dehydrocholesterol reductase | -2.23 | 0.002225403 |
| Cdc25b | NM_023117 | cell division cycle 25 homolog B (S. cerevisiae) | -2.22 | 0.01731473 |
| 2410003B16Rik | XM_204283 | RIKEN cDNA 2410003B16 gene | -2.21 | 0.00342568 |
| 4432405B04Rik | NM_026486 | RIKEN cDNA 4432405B04 gene | -2.20 | 0.00036279 |
| Ptpru | NM_011214 | protein tyrosine phosphatase, receptor type, U | -2.20 | 0.00088875 |
| Cnp1 | NM_009923 | cyclic nucleotide phosphodiesterase 1 | -2.20 | 0.00142575 |
| Tspan1 | NM_133681 | tetraspan 1 | -2.20 | 0.00040954 |
| Fads1 | NM_146094 | fatty acid desaturase 1 | -2.19 | 0.00090774 |
| Eppk1 | NM_144848 | epiplakin 1 | -2.18 | 0.00117225 |
| Spsb1 | NM_029035 | splA/ryanodine receptor domain and SOCS box containing 1 | -2.17 | 0.00038232 |
| Ttc12 | NM_172770 | tetratricopeptide repeat domain 12 | -2.17 | 0.00095151 |
| H6pd | NM_173371 | hexose-6-phosphate dehydrogenase (glucose 1-dehydrogenase) | -2.16 | 0.00071079 |
| Anxa8 | NM_013473 | annexin A8 | -2.15 | 0.00085333 |
| Tcf2 | NM_009330 | transcription factor 2 | -2.14 | 0.00076234 |
| 2700055K07Rik | NM_026481 | RIKEN cDNA 2700055K07 gene | -2.13 | 0.00110917 |
| 4833401D15Rik | NM_175030 | RIKEN cDNA 4833401D15 gene | -2.12 | 0.00100947 |
| Ntf3 | NM_008742 | neurotrophin 3 | -2.12 | 0.00380362 |
| Tsnaxip1 | NM_024445 | translin-associated factor X (Tsnax) interacting protein 1 | -2.12 | 0.00083862 |
| 2700059L22Rik | NM_028300 | RIKEN cDNA 2700059L22 gene | -2.11 | 0.00115378 |
| Hras1 | NM_008284 | Harvey rat sarcoma virus oncogene 1 | -2.11 | 0.00432954 |
| Armc4 | XM_619518 | armadillo repeat containing 4 | -2.11 | 0.00076781 |
| Scnn1g | NM_011326 | sodium channel, nonvoltage-gated 1 gamma | -2.11 | 0.00128211 |
| 2810432L12Rik | NM_025944 | RIKEN cDNA 2810432L12 gene | -2.10 | 0.00127702 |
| Ngef | NM_019867 | neuronal guanine nucleotide exchange factor | -2.10 | 0.00116516 |
| 4632415K11Rik | NM_028883 | RIKEN cDNA 4632415K11 gene | -2.09 | 0.00081809 |
| Ggtla1 | NM_011820 | gamma-glutamyltransferase-like activity 1 | -2.09 | 0.03510263 |
| BC025519 | NM_001030014 | cDNA sequence BC025519 | -2.09 | 0.0008995 |
| Porcn | NM_016913 | porcupine homolog (Drosophila) | -2.09 | 0.00126618 |
| Prss22 | NM_133731 | protease, serine, 22 | -2.09 | 0.00132308 |
| Clic6 | NM_172469 | chloride intracellular channel 6 | -2.09 | 0.00425633 |
| Gstm2 | NM_008183 | glutathione S-transferase, mu 2 | -2.08 | 0.00774132 |
| C030048H21Rik |  | RIKEN cDNA C030048H21 gene | -2.08 | 0.00834433 |
| Rnd2 | NM_009708 | Rho family GTPase 2 | -2.06 | 0.00055258 |
| Aqp1 | NM_007472 | aquaporin 1 | -2.06 | 0.00222391 |
| MGI:2446326 | NM_172205 | suprabasin | -2.05 | 0.0011231 |
| 2410022L05Rik | NM_025556 | RIKEN cDNA 2410022L05 gene | -2.04 | 0.0009425 |
| Htra1 | NM_019564 | HtrA serine peptidase 1 | -2.04 | 0.00137495 |
| Elovl1 | NM_019422 | elongation of very long chain fatty acids (FEN1/Elo2, SUR4/Elo3, yeast)-like 1 | -2.04 | 0.00079489 |
| AI467606 | NM_178901 | expressed sequence AI467606 | -2.03 | 0.01298716 |
| Qscn6 | NM_023268 | quiescin Q6 | -2.03 | 0.01011586 |
| Bet1l | NM_018742 | blocked early in transport 1 homolog (S. cerevisiae)-like | -2.02 | 0.0008637 |
| Fstl1 | NM_008047 | follistatin-like 1 | -2.02 | 0.01405527 |
| 1110004B13Rik | NM_025838 | RIKEN cDNA 1110004B13 gene | -2.01 | 0.00073945 |
| Gp1bb | NM_001001999 | glycoprotein Ib, beta polypeptide | -2.01 | 0.00136696 |
| Rptn | NM_009100 | repetin | -2.01 | 0.00080859 |
| Cnn3 | NM_028044 | calponin 3, acidic | -2.00 | 0.01064599 |
| Cyp4b1 | NM_007823 | cytochrome P450, family 4, subfamily b, polypeptide 1 | -2.00 | 0.0009322 |
| Dnmt1 | NM_010066 | DNA methyltransferase (cytosine-5) 1 | -2.00 | 0.02798627 |
| Ripk4 | NM_023663 | receptor-interacting serine-threonine kinase 4 | -2.00 | 0.01424578 |
| Foxa2 | NM_010446 | forkhead box A2 | -1.99 | 0.00201141 |
| 1700094D03Rik |  | RIKEN cDNA 1700094D03 gene | -1.98 | 0.00591935 |
| Sdh1 | NM_146126 | sorbitol dehydrogenase 1 | -1.98 | 0.01861419 |
| 4930414L22Rik |  | RIKEN cDNA 4930414L22 gene | -1.97 | 0.011903 |
| Jak3 | NM_010589 | Janus kinase 3 | -1.97 | 0.01125858 |
| Ddr1 | NM_007584 | discoidin domain receptor family, member 1 | -1.97 | 0.00090801 |
| Enpp2 | NM_015744 | ectonucleotide pyrophosphatase/phosphodiesterase 2 | -1.96 | 0.00763791 |
| Syvn1 | NM_028769 | synovial apoptosis inhibitor 1, synoviolin | -1.95 | 0.00984991 |
| Ubxd3 | NM_178671 | UBX domain containing 3 | -1.94 | 0.00596112 |
| Tcf3 | NM_009332 | transcription factor 3 | -1.93 | 0.00427106 |
| Haghl | NM_026897 | hydroxyacylglutathione hydrolase-like | -1.93 | 0.00179276 |
| Atp9a | NM_015731 | ATPase, class II, type 9A | -1.92 | 0.00694053 |
| Fdft1 | NM_010191 | farnesyl diphosphate farnesyl transferase 1 | -1.92 | 0.01399138 |
| Gys3 | NM_008195 | glycogen synthase 3, brain | -1.92 | 0.005222 |
| 1700019G17Rik | NM_029331 | RIKEN cDNA 1700019G17 gene | -1.91 | 0.0008015 |
| Dgcr6 | NM_010047 | DiGeorge syndrome critical region gene 6 | -1.91 | 0.00178389 |
| Hk1 | NM_010438 | hexokinase 1 | -1.90 | 0.00743006 |
| Rsnl2 | NM_030179 | restin-like 2 | -1.90 | 0.0045579 |
| C1r | NM_023143 | complement component 1, r subcomponent | -1.90 | 0.00636071 |
| Adora2b | NM_007413 | adenosine A2b receptor | -1.89 | 0.00416345 |
| Slc7a4 | NM_144852 | solute carrier family 7 (cationic amino acid transporter, y+ sys | -1.89 | 0.00580157 |
| 1110006I15Rik | NM_134142 | RIKEN cDNA 1110006I15 gene | -1.88 | 0.00399446 |
| AA409316 | NM_134087 | expressed sequence AA409316 | -1.88 | 0.00176324 |
| C79267 | NM_183148 | expressed sequence C79267 | -1.88 | 0.00691933 |
| Ivd | NM_019826 | isovaleryl coenzyme A dehydrogenase | -1.88 | 0.0076397 |
| Lss | NM_146006 | lanosterol synthase | -1.87 | 0.00335637 |
| Pik3r4 | XM_135116 | phosphatidylinositol 3 kinase, regulatory subunit, polypeptide 4 | -1.86 | 0.0192324 |
| Trub2 | NM_145520 | TruB pseudouridine (psi) synthase homolog 2 (E. coli) | -1.86 | 0.00823064 |
| 2610002J02Rik | XM_131827 | RIKEN cDNA 2610002J02 gene | -1.85 | 0.00474342 |
| Biklk | NM_007546 | Bcl2-interacting killer-like | -1.85 | 0.00459892 |
| Pex6 | NM_145488 | peroxisomal biogenesis factor 6 | -1.85 | 0.02352508 |
| Mvk | NM_023556 | mevalonate kinase | -1.85 | 0.007145721 |
| 6330579B17Rik | NM_026494 | RIKEN cDNA 6330579B17 gene | -1.84 | 0.00982913 |
| Ptges | NM_022415 | prostaglandin E synthase | -1.84 | 0.00423049 |
| Pvrl2 | NM_008990 | poliovirus receptor-related 2 | -1.82 | 0.01495085 |
| Rapgef3 | NM_144850 | Rap guanine nucleotide exchange factor (GEF) 3 | -1.82 | 0.00384414 |
| Aldh7a1 | NM_138600 | aldehyde dehydrogenase family 7, member A1 | -1.82 | 0.00823766 |
| Ascc2 | NM_029291 | activating signal cointegrator 1 complex subunit 2 | -1.81 | 0.00216559 |
| Fbxw8 | NM_172721 | F-box and WD-40 domain protein 8 | -1.81 | 0.01212724 |
| Pfkl | NM_008826 | phosphofructokinase, liver, B-type | -1.81 | 0.01302797 |
| Hadh2 | NM_016763 | hydroxyacyl-Coenzyme A dehydrogenase type II | -1.80 | 0.00672367 |
| AA986860 | NM_177604 | expressed sequence AA986860 | -1.80 | 0.00827553 |
| Gss | NM_008180 | glutathione synthetase | -1.79 | 0.00751693 |
| Gja1 | NM_010288 | gap junction membrane channel protein alpha 1 | -1.79 | 0.00152648 |
| Pmvk | NM_026784 | phosphomevalonate kinase | -1.79 | 0.00894554 |
| Dos | NM_011492 | downstream of Stk11 | -1.78 | 0.00171746 |
| Fbln2 | NM_007992 | fibulin 2 | -1.78 | 0.00133807 |
| Fdps | NM_134469 | farnesyl diphosphate synthetase | -1.78 | 0.001560758 |
| Entpd4 | NM_026174 | ectonucleoside triphosphate diphosphohydrolase 4 | -1.77 | 0.00976794 |
| Pcbp4 | NM_021567 | poly(rC) binding protein 4 | -1.77 | 0.01421208 |
| Wdr10 | NM_031177 | WD repeat domain 10 | -1.76 | 0.009062 |
| Il11ra1 | NM_010549 | interleukin 11 receptor, alpha chain 1 | -1.76 | 0.00695019 |
| Ovol1 | NM_019935 | OVO homolog-like 1 (Drosophila) | -1.75 | 0.00430022 |
| Pias4 | NM_021501 | protein inhibitor of activated STAT, 4 | -1.75 | 0.01573472 |
| Mrpl44 | NM_026970 | mitochondrial ribosomal protein L44 | -1.74 | 0.0039582 |
| Pfn2 | NM_019410 | profilin 2 | -1.74 | 0.00305322 |
| Bcr | XM_125706 | breakpoint cluster region homolog | -1.74 | 0.00231005 |
| Ptov1 | NM_133949 | prostate tumor over expressed gene 1 | -1.74 | 0.0092049 |
| Stat3 | NM_011486 | signal transducer and activator of transcription 3 | -1.74 | 0.00327297 |
| Commd9 | NM_029635 | COMM domain containing 9 | -1.74 | 0.00505379 |
| Ruvbl1 | NM_019685 | RuvB-like protein 1 | -1.73 | 0.00725731 |
| Cds2* | NM_138651 | CDP-diacylglycerol synthase (phosphatidate cytidylyltransferase) | -1.72 | 0.00486378 |
| Phc2 | NM_018774 | polyhomeotic-like 2 (Drosophila) | -1.72 | 0.00170314 |
| Ccbl1 | NM_172404 | cysteine conjugate-beta lyase 1 | -1.72 | 0.00241772 |
| 2410004H02Rik | NM_145954 | RIKEN cDNA 2410004H02 gene | -1.72 | 0.00834441 |
| Hist2h2bb | NM_175666 | histone 2, H2bb | -1.72 | 0.0311399 |
| Pcx | NM_008797 | pyruvate carboxylase | -1.71 | 0.02623397 |
| Tcta | NM_133986 | T-cell leukemia translocation altered gene | -1.71 | 0.00680362 |
| Prkcdbp | NM_028444 | protein kinase C, delta binding protein | -1.71 | 0.00266781 |
| Snapc2 | NM_133968 | small nuclear RNA activating complex, polypeptide 2 | -1.71 | 0.00133979 |
| Pgd | NM_025801 | phosphogluconate dehydrogenase | -1.71 | 0.00903675 |
| Acox2* | NM_053115 | acyl-Coenzyme A oxidase 2, branched chain | -1.70 | 0.00559243 |
| Tkt | NM_009388 | transketolase | -1.70 | 0.01588153 |
| Ccnd3 | NM_007632 | cyclin D3 | -1.70 | 0.00269707 |
| Fzd3 | NM_021458 | frizzled homolog 3 (Drosophila) | -1.70 | 0.00226268 |
| Irf1 | NM_008390 | interferon regulatory factor 1 | -1.70 | 0.00198459 |
| 0610025L06Rik | NM_172397 | RIKEN cDNA 0610025L06 gene | -1.69 | 0.00637696 |
| Cpt2 | NM_009949 | carnitine palmitoyltransferase 2 | -1.69 | 0.01994915 |
| Hdc | NM_008230 | histidine decarboxylase | -1.69 | 0.01504142 |
| MGI:1353606 | NM_013929 | Cd27 binding protein (Hindu God of destruction) | -1.69 | 0.00278666 |
| Rabl4 | NM_025931 | RAB, member of RAS oncogene family-like 4 | -1.69 | 0.03241024 |
| Srebf1* | NM_011480 | sterol regulatory element binding factor 1 | -1.69 | 0.02462055 |
| Rshl2 | NM_025789 | radial spokehead-like 2 | -1.69 | 0.00510954 |
| Fzd2 | NM_020510 | frizzled homolog 2 (Drosophila) | -1.69 | 0.00205101 |
| Akr7a5 | NM_025337 | aldo-keto reductase family 7, member A5 (aflatoxin aldehyde redu | -1.68 | 0.01160716 |
| D10Bwg1364e | NM_145421 | DNA segment, Chr 10, Brigham & Women's Genetics 1364 expressed | -1.68 | 0.01714149 |
| Traf2 | NM_009422 | Tnf receptor-associated factor 2 | -1.68 | 0.00277281 |
| Bdkrb2 | NM_009747 | Bradykinin receptor, beta 2 (Bdkrb2), mRNA | -1.67 | 0.00343431 |
| Fzd7 | NM_008057 | frizzled homolog 7 (Drosophila) | -1.66 | 0.01727846 |
| AI325464 | NM_178882 | expressed sequence AI325464 | -1.66 | 0.00375267 |
| Hp | NM_017370 | haptoglobin | -1.65 | 0.00775803 |
| Apbb1 | NM_009685 | amyloid beta (A4) precursor protein-binding, family B, member 1 | -1.65 | 0.0211471 |
| Ddit3 | NM_007837 | DNA-damage inducible transcript 3 | -1.65 | 0.0393635 |
| Mkrn1 | NM_018810 | makorin, ring finger protein, 1 | -1.65 | 0.00345509 |
| Ncdn | NM_011986 | neurochondrin | -1.65 | 0.01996902 |
| Ndufs8 | NM_144870 | NADH dehydrogenase (ubiquinone) Fe-S protein 8 | -1.65 | 0.03769662 |
| Cd151 | NM_009842 | CD151 antigen | -1.65 | 0.00744863 |
| Hes6 | NM_019479 | hairy and enhancer of split 6 (Drosophila) | -1.65 | 0.00475562 |
| Coasy | NM_027896 | Coenzyme A synthase | -1.65 | 0.00618513 |
| Pigq | NM_011822 | phosphatidylinositol glycan, class Q | -1.64 | 0.00423224 |
| 1110001K21Rik | NM_025839 | RIKEN cDNA 1110001K21 gene | -1.64 | 0.00881174 |
| Ppp1r16a | NM_033371 | protein phosphatase 1, regulatory (inhibitor) subunit 16A | -1.64 | 0.00508042 |
| 1110061L23Rik | NM_029406 | RIKEN cDNA 1110061L23 gene | -1.64 | 0.00310037 |
| Acaa1 | NM_130864 | acetyl-Coenzyme A acyltransferase 1 | -1.64 | 0.01966365 |
| Trib3 | NM_144554 | tribbles homolog 3 (Drosophila) | -1.63 | 0.00295239 |
| Bzrp | NM_009775 | benzodiazepine receptor, peripheral | -1.63 | 0.00466509 |
| Pik3r2 | NM_008841 | phosphatidylinositol 3-kinase, regulatory subunit, polypeptide 2 | -1.63 | 0.01219034 |
| Wnt4 | NM_009523 | wingless-related MMTV integration site 4 | -1.63 | 0.01397718 |
| Mrpl28 | NM_024227 | mitochondrial ribosomal protein L28 | -1.63 | 0.00478075 |
| Fliih | NM_022009 | flightless I homolog (Drosophila) | -1.62 | 0.00495257 |
| Mrps24 | NM_026080 | mitochondrial ribosomal protein S24 | -1.62 | 0.00492707 |
| 1700013F07Rik | XM_131080 | RIKEN cDNA 1700013F07 gene | -1.62 | 0.00402697 |
| Eif2b2 | NM_145445 | eukaryotic translation initiation factor 2B, subunit 2 beta | -1.61 | 0.00699843 |
| Park7 | NM_020569 | Parkinson disease (autosomal recessive, early onset) 7 | -1.61 | 0.00948318 |
| Trp53bp1 | NM_013735 | transformation related protein 53 binding protein 1 | -1.61 | 0.00270436 |
| Rbx1 | NM_019712 | ring-box 1 | -1.61 | 0.00352889 |
| Sqle | NM_009270 | squalene epoxidase | -1.61 | 0.00851955 |
| 5730472N09Rik | NM_175392 | RIKEN cDNA 5730472N09 gene | -1.60 | 0.0090641 |
| Psmf1 | NM_144889 | proteasome (prosome, macropain) inhibitor subunit 1 | -1.60 | 0.00202072 |
| Map1lc3a | NM_025735 | microtubule-associated protein 1 light chain 3 alpha | -1.60 | 0.0032941 |
| Smpd1 | NM_011421 | sphingomyelin phosphodiesterase 1, acid lysosomal | -1.60 | 0.02831757 |
| Apex1 | NM_009687 | apurinic/apyrimidinic endonuclease 1 | -1.59 | 0.00796302 |
| Abca3* | NM_013855 | ATP-binding cassette, sub-family A (ABC1), member 3 | -1.59 | 0.00389322 |
| Cdc91l1 | NM_001004721 | cell division cycle 91-like 1 (S. cerevisiae) | -1.59 | 0.00411615 |
| Prp19 | NM_134129 | PRP19/PSO4 homolog (S. cerevisiae) | -1.59 | 0.01638466 |
| Ptdss2 | NM_013782 | phosphatidylserine synthase 2 | -1.59 | 0.01514253 |
| Tgfb2 | NM_009367 | transforming growth factor, beta 2 | -1.59 | 0.01433536 |
| Txnrd3 | NM_153162 | thioredoxin reductase 3 | -1.59 | 0.01670849 |
| Pbx2 | NM_017463 | pre B-cell leukemia transcription factor 2 | -1.59 | 0.00481006 |
| Pbp | NM_018858 | phosphatidylethanolamine binding protein | -1.58 | 0.00302863 |
| Akt2* | NM_007434 | thymoma viral proto-oncogene 2 | -1.58 | 0.00885546 |
| Acat2 | NM_009338 | acetyl-Coenzyme A acetyltransferase 2 | -1.58 | 0.02169944 |
| Vdac1 | NM_010162 | voltage-dependent anion channel 1 | -1.58 | 0.00451027 |
| G630024C07Rik | NM_177362 | RIKEN cDNA G630024C07 gene | -1.58 | 0.01142866 |
| Ndrg1 | NM_010884 | N-myc downstream regulated gene 1 | -1.58 | 0.01006218 |
| Pmm1 | NM_013872 | phosphomannomutase 1 | -1.58 | 0.01078206 |
| Scap* | NM_001001144 | SREBP cleavage activating protein | -1.58 | 0.00513467 |
| Gsn | NM_146120 | gelsolin | -1.58 | 0.00607971 |
| Scd2 | NM_009128 | stearoyl-Coenzyme A desaturase 2 | -1.58 | 0.003546886 |
| 1110015K06Rik | NM_026748 | RIKEN cDNA 1110015K06 gene | -1.57 | 0.00600796 |
| 1700012G19Rik | NM_025954 | RIKEN cDNA 1700012G19 gene | -1.57 | 0.02604776 |
| 1700016F23Rik | NM_029064 | RIKEN cDNA 1700016F23 gene | -1.57 | 0.01624784 |
| Bad | NM_007522 | Bcl-associated death promoter | -1.57 | 0.00789347 |
| Fasn* | NM_007988 | fatty acid synthase | -1.57 | 0.01878787 |
| Socs3 | NM_007707 | suppressor of cytokine signaling 3 | -1.57 | 0.01098529 |
| Acads | NM_007383 | acyl-Coenzyme A dehydrogenase, short chain | -1.56 | 0.02664521 |
| D11Ertd333e | NM_026542 | DNA segment, Chr 11, ERATO Doi 333, expressed | -1.56 | 0.00912795 |
| Icam1 | NM_010493 | intercellular adhesion molecule | -1.56 | 0.00485785 |
| Cdc25a | NM_007658 | cell division cycle 25 homolog A (S. cerevisiae) | -1.55 | 0.00999652 |
| Evpl | NM_025276 | envoplakin | -1.55 | 0.00678602 |
| Mmrn2 | NM_153127 | multimerin 2 | -1.55 | 0.01832475 |
| Pxmp4 | NM_021534 | peroxisomal membrane protein 4 | -1.54 | 0.00443126 |
| Bcl2l1* | NM_009743 | Bcl2-like 1 | -1.54 | 0.02705617 |
| Dhx30 | NM_133347 | DEAH (Asp-Glu-Ala-His) box polypeptide 30 | -1.54 | 0.00647701 |
| Gpam* | NM_008149 | glycerol-3-phosphate acyltransferase, mitochondrial | -1.54 | 0.03471 |
| Ldlr |  | Low density lipoprotein receptor | -1.54 | 0.01443419 |
| Cdipt | NM_026638 | CDP-diacylglycerol--inositol 3-phosphatidyltransferase (phosphat | -1.54 | 0.00836905 |
| Ldh2 | NM_008492 | lactate dehydrogenase 2, B chain | -1.53 | 0.00434614 |
| Srebf2* | NM_033218 | sterol regulatory element binding factor 2 | -1.50 | 0.024111089 |
| Acaca | NM_133360 | acetyl-Coenzyme A carboxylase alpha | -1.50 | 0.008945544 |
| Fxr1h | NM_008053 | fragile X mental retardation gene 1, autosomal homolog | 1.51 | 0.01424753 |
| Mgat2 | NM_146035 | mannoside acetylglucosaminyltransferase 2 | 1.51 | 0.01151414 |
| Trio |  | RIKEN cDNA 6720464I07 gene | 1.51 | 0.00589085 |
| Smad4 | NM_008540 | MAD homolog 4 (Drosophila) | 1.52 | 0.00430701 |
| App | --- | amyloid beta (A4) precursor protein | 1.52 | 0.00459501 |
| Bcl2 | NM_027395 | B-cell leukemia/lymphoma 2 | 1.52 | 0.00634939 |
| 1110059H15Rik | XM_485005 | RIKEN cDNA 1110059H15 gene | 1.53 | 0.01673229 |
| Cbfb | NM_022309 | Core binding factor beta | 1.54 | 0.0054786 |
| 2900024P20Rik | XM_484214 | RIKEN cDNA 2900024P20 gene | 1.55 | 0.02208962 |
| Stk39 | NM_016866 | serine/threonine kinase 39, STE20/SPS1 homolog (yeast) | 1.56 | 0.00815653 |
| Emb | NM_010330 | embigin | 1.56 | 0.00810371 |
| Itga9 | NM_133721 | integrin alpha 9 | 1.56 | 0.00667214 |
| D2Bwg1356e | XM_130523 | DNA segment, Chr 2, Brigham & Women's Genetics 1356 expressed | 1.56 | 0.00632903 |
| Ddx5 | NM_007840 | DEAD (Asp-Glu-Ala-Asp) box polypeptide 5 | 1.57 | 0.00933447 |
| Shc1 | NM_011368 | src homology 2 domain-containing transforming protein C1 | 1.57 | 0.00735092 |
| Vldlr | NM_013703 | very low density lipoprotein receptor | 1.57 | 0.00749333 |
| Pura | NM_008989 | purine rich element binding protein A | 1.58 | 0.00605199 |
| Cebpg | NM_009884 | CCAAT/enhancer binding protein (C/EBP), gamma | 1.58 | 0.02531788 |
| D8Ertd594e | NM_133791 | DNA segment, Chr 8, ERATO Doi 594, expressed | 1.59 | 0.00859813 |
| Sos2 | XM_127051 | Son of sevenless homolog 2 (Drosophila) | 1.59 | 0.00619166 |
| 1190002N15Rik | XM_147036 | RIKEN cDNA 1190002N15 gene | 1.59 | 0.02074877 |
| Arfrp2 | NM_172595 | ADP-ribosylation factor related protein 2 | 1.59 | 0.00273955 |
| B130008E12Rik | XM_137316 | RIKEN cDNA B130008E12 gene | 1.59 | 0.01142422 |
| Stam2 | NM_019667 | signal transducing adaptor molecule (SH3 domain and ITAM motif) 2 | 1.60 | 0.0099082 |
| E430027O22Rik | XM_129248 | RIKEN cDNA E430027O22 gene | 1.60 | 0.00987662 |
| Ube2b | NM_009458 | ubiquitin-conjugating enzyme E2B, RAD6 homology (S. cerevisiae) | 1.60 | 0.01115917 |
| 1700034M03Rik | NM_024260 | RIKEN cDNA 1700034M03 gene | 1.60 | 0.00519569 |
| Braf | XM_355754 | Braf transforming gene | 1.61 | 0.00565128 |
| 2610312B22Rik | NM_026934 | RIKEN cDNA 2610312B22 gene | 1.61 | 0.0230792 |
| Smad3 | NM_016769 | MAD homolog 3 (Drosophila) | 1.61 | 0.02009284 |
| Dhx36 | NM_028136 | DEAH (Asp-Glu-Ala-His) box polypeptide 36 | 1.61 | 0.00446709 |
| 2700078E11Rik | NM_030197 | RIKEN cDNA 2700078E11 gene | 1.62 | 0.00524436 |
| Asb13 | NM_080857 | ankyrin repeat and SOCS box-containing protein 13 | 1.62 | 0.00478431 |
| Ascc3 | XM_125617 | activating signal cointegrator 1 complex subunit 3 | 1.62 | 0.02100641 |
| Mll3 | XM_355579 | myeloid/lymphoid or mixed-lineage leukemia 3 | 1.63 | 0.03444127 |
| AW061290 | NM_201361 | expressed sequence AW061290 | 1.63 | 0.00982529 |
| Lcp1 | NM_008879 | lymphocyte cytosolic protein 1 | 1.63 | 0.00277669 |
| Kcnip4 | NM_030265 | Kv channel interacting protein 4 | 1.63 | 0.0095809 |
| Gca | NM_145523 | grancalcin | 1.64 | 0.00640343 |
| Ubxd4 | NM_145441 | UBX domain containing 4 | 1.64 | 0.00622193 |
| Gna12 | NM_010302 | guanine nucleotide binding protein, alpha 12 | 1.64 | 0.00566119 |
| Mxi1 | NM_001008542 | Max interacting protein 1 | 1.64 | 0.00328318 |
| Rap1a | NM_145541 | RAS-related protein-1a | 1.64 | 0.0091745 |
| 4933403F05Rik | NM_153794 | RIKEN cDNA 4933403F05 gene | 1.65 | 0.00072338 |
| Prps2 | NM_026662 | phosphoribosyl pyrophosphate synthetase 2 | 1.65 | 0.00715336 |
| Mcl1 | NM_008562 | myeloid cell leukemia sequence 1 | 1.65 | 0.01086875 |
| Tbk1 | NM_019786 | TANK-binding kinase 1 | 1.65 | 0.00957256 |
| Dnajc3 | NM_008929 | DnaJ (Hsp40) homolog, subfamily C, member 3 | 1.66 | 0.01652498 |
| Laf4l | NM_033565 | lymphoid nuclear protein related to AF4-like | 1.66 | 0.00248686 |
| Cept1 | NM_133869 | Choline/ethanolaminephosphotransferase 1 | 1.66 | 0.00715168 |
| Nap1l1 | NM_015781 | nucleosome assembly protein 1-like 1 | 1.66 | 0.00854609 |
| Sfrs2ip | XM_128178 | splicing factor, arginine/serine-rich 2, interacting protein | 1.66 | 0.00430255 |
| Ncoa3 | NM_008679 | Nuclear receptor coactivator 3 (Ncoa3), mRNA | 1.67 | 0.00456321 |
| D10Ertd438e | NM_030250 | DNA segment, Chr 10, ERATO Doi 438, expressed | 1.67 | 0.0033791 |
| 1600012F09Rik | NM_025904 | RIKEN cDNA 1600012F09 gene | 1.67 | 0.03723465 |
| Mapk14 | NM_011951 | mitogen activated protein kinase 14 | 1.68 | 0.00537615 |
| Hnrpf | NM_133834 | heterogeneous nuclear ribonucleoprotein F | 1.68 | 0.03566471 |
| Pitpnc1 | NM_145823 | phosphatidylinositol transfer protein, cytoplasmic 1 | 1.68 | 0.00735937 |
| Asf1a | NM_025541 | ASF1 anti-silencing function 1 homolog A (S. cerevisiae) | 1.69 | 0.01503741 |
| Cdc37l1 | NM_025950 | cell division cycle 37 homolog (S. cerevisiae)-like 1 | 1.69 | 0.01933272 |
| Csf2rb2 | NM_007781 | colony stimulating factor 2 receptor, beta 2, low-affinity (gran | 1.69 | 0.01170524 |
| Trp53inp1 | NM_021897 | transformation related protein 53 inducible nuclear protein 1 | 1.69 | 0.00134469 |
| Narg1 | NM_053089 | NMDA receptor-regulated gene 1 | 1.69 | 0.00360898 |
| Rala | NM_019491 | v-ral simian leukemia viral oncogene homolog A (ras related) | 1.69 | 0.00688759 |
| Srpk2 | NM_009274 | serine/arginine-rich protein specific kinase 2 | 1.69 | 0.00663119 |
| Mynn | NM_030557 | myoneurin | 1.70 | 0.0028485 |
| Hrb | NM_010472 | HIV-1 Rev binding protein | 1.70 | 0.00481633 |
| LOC278757 | NM_177596 | similar to hypothetical protein 6720451E15 | 1.70 | 0.00583216 |
| Nupl1 | NM_170591 | nucleoporin like 1 | 1.70 | 0.01807998 |
| Rab1 | NM_008996 | RAB1, member RAS oncogene family | 1.70 | 0.00375456 |
| 2010309L07Rik |  | RIKEN cDNA 2010309L07 gene | 1.70 | 0.00848976 |
| Acp1 | NM_021330 | acid phosphatase 1, soluble | 1.71 | 0.00646874 |
| Il6st | NM_010560 | interleukin 6 signal transducer | 1.71 | 0.0023543 |
| Ugp2 | NM_139297 | UDP-glucose pyrophosphorylase 2 | 1.71 | 0.00139028 |
| Itpr1 | NM_010585 | Inositol 1,4,5-triphosphate receptor 1 | 1.71 | 0.00451517 |
| Myo1b | NM_010863 | myosin IB | 1.71 | 0.01642977 |
| Myo5a | NM_010864 | myosin Va | 1.71 | 0.01237677 |
| Ibrdc3 | XM_204030 | IBR domain containing 3 | 1.71 | 0.001091 |
| Map2k4 | NM_009157 | mitogen activated protein kinase kinase 4 | 1.72 | 0.00844648 |
| 5031439A09Rik | NM_026582 | RIKEN cDNA 5031439A09 gene | 1.72 | 0.00207338 |
| Eif3s1 | NM_144545 | eukaryotic translation initiation factor 3, subunit 1 alpha | 1.73 | 0.0126713 |
| Mtdh | NM_026002 | Metadherin | 1.73 | 0.01138181 |
| Snx13 | XM_126867 | sorting nexin 13 | 1.73 | 0.00302226 |
| Tmem33 | NM_028975 | transmembrane protein 33 | 1.73 | 0.00697018 |
| Cnot6l | NM_144910 | CCR4-NOT transcription complex, subunit 6-like | 1.73 | 0.00277091 |
| Cxcl4 | NM_019932 | chemokine (C-X-C motif) ligand 4 | 1.73 | 0.00366838 |
| Rif1 | NM_175238 | Rap1 interacting factor 1 homolog (yeast) | 1.73 | 0.00079884 |
| 2610318G18Rik | NM_026013 | RIKEN cDNA 2610318G18 gene | 1.74 | 0.00959153 |
| Cxcl10 | NM_021274 | chemokine (C-X-C motif) ligand 10 | 1.74 | 0.03041021 |
| Prei3 | NM_025283 | preimplantation protein 3 | 1.74 | 0.01131213 |
| Rbm6 | NM_011251 | RNA binding motif protein 6 | 1.74 | 0.0154225 |
| Top2b | NM_009409 | topoisomerase (DNA) II beta | 1.74 | 0.01320592 |
| Schip1 | NM_013928 | Schwannomin interacting protein 1 | 1.75 | 0.00498195 |
| Irf2bp2 | XM_284454 | interferon regulatory factor 2 binding protein 2 | 1.75 | 0.0025428 |
| Herc4 | NM_026101 | hect domain and RLD 4 | 1.75 | 0.0158804 |
| Ccnd2 | NM_009829 | cyclin D2 | 1.75 | 0.00827051 |
| Cul1 | NM_012042 | cullin 1 | 1.76 | 0.00278295 |
| Grasp | NM_019518 | GRP1 (general receptor for phosphoinositides 1)-associated scaff | 1.76 | 0.00240319 |
| Tnfrsf13c | NM_028075 | tumor necrosis factor receptor superfamily, member 13c | 1.76 | 0.00250698 |
| Crkl | NM_007764 | v-crk sarcoma virus CT10 oncogene homolog (avian)-like | 1.76 | 0.00139905 |
| Arl6ip6 | NM_022989 | ADP-ribosylation factor-like 6 interacting protein 6 | 1.76 | 0.03364739 |
| Ifngr2 | NM_008338 | interferon gamma receptor 2 | 1.76 | 0.0179967 |
| Pfkp | NM_019703 | phosphofructokinase, platelet | 1.76 | 0.00716996 |
| 8430423A01Rik | NM_175294 | RIKEN cDNA 8430423A01 gene | 1.76 | 0.00133231 |
| Ttn | XM_130322 | titin | 1.76 | 0.009163 |
| Gnpnat1 | NM_019425 | glucosamine-phosphate N-acetyltransferase 1 | 1.77 | 0.010702 |
| Nol8 | XM_484255 | nucleolar protein 8 | 1.77 | 0.00173104 |
| Xrn2 | NM_011917 | 5'-3' exoribonuclease 2 | 1.77 | 0.00387122 |
| Kpna4 | NM_008467 | karyopherin (importin) alpha 4 | 1.77 | 0.00317292 |
| Pdzk8 | NM_172523 | PDZ domain containing 8 | 1.78 | 0.00191304 |
| Zbtb10 | XM_485202 | zinc finger and BTB domain containing 10 | 1.78 | 0.00131472 |
| Ddhd1 | NM_176845 | DDHD domain containing 1 | 1.78 | 0.02352447 |
| Kpna1 | NM_008465 | karyopherin (importin) alpha 1 | 1.78 | 0.00606674 |
| Sfrs6 | NM_026499 | splicing factor, arginine/serine-rich 6 | 1.78 | 0.01131386 |
| Cul4b | NM_028288 | cullin 4B | 1.78 | 0.00432823 |
| Phf3 | XM_129836 | PHD finger protein 3 | 1.78 | 0.00952198 |
| Il18rap | NM_010553 | interleukin 18 receptor accessory protein | 1.79 | 0.00644148 |
| Rps6kb1 | NM_028259 | ribosomal protein S6 kinase, polypeptide 1 | 1.79 | 0.00245163 |
| Itgb1 | NM_010578 | integrin beta 1 (fibronectin receptor beta) | 1.79 | 0.00684975 |
| Cd80 | NM_009855 | CD80 antigen | 1.79 | 0.00128285 |
| Usp12 | NM_011669 | ubiquitin specific peptidase 12 | 1.79 | 0.00119512 |
| Csf1r | NM_007779 | colony stimulating factor 1 receptor | 1.80 | 0.03356391 |
| Elf1 | NM_007920 | E74-like factor 1 | 1.80 | 0.00738983 |
| Mbnl1 | NM_020007 | Muscleblind-like 1 (Drosophila) | 1.80 | 0.01534565 |
| Rsn | NM_019765 | restin (Reed-Steinberg cell-expressed intermediate filament-asso | 1.80 | 0.00183235 |
| Jag1 | NM_013822 | jagged 1 | 1.80 | 0.0039105 |
| Nfia | NM_177176 | nuclear factor I/A | 1.80 | 0.00768242 |
| Aebp2 | NM_001005605 | AE binding protein 2 | 1.81 | 0.00276196 |
| Arf2 | NM_007477 | ADP-ribosylation factor 2 | 1.81 | 0.02389058 |
| D5Ertd585e | NM_027922 | DNA segment, Chr 5, ERATO Doi 585, expressed | 1.81 | 0.01115977 |
| Epha4 | NM_007936 | Eph receptor A4 | 1.81 | 0.00573233 |
| Homer1 | NM_011982 | homer homolog 1 (Drosophila) | 1.81 | 0.00774039 |
| Mitf | NM_008601 | microphthalmia-associated transcription factor | 1.81 | 0.00144649 |
| A230035H12Rik |  | RIKEN cDNA A230035H12 gene | 1.81 | 0.00408075 |
| Chic2 | NM_028850 | cysteine-rich hydrophobic domain 2 | 1.81 | 0.0074575 |
| Ubap2l | NM_028475 | Ubiquitin associated protein 2-like (Ubap2l), transcript variant 2, mRNA | 1.81 | 0.00103139 |
| 4933421G18Rik | NM_198293 | RIKEN cDNA 4933421G18 gene | 1.82 | 0.00185572 |
| 0610041E09Rik | NM_025335 | RIKEN cDNA 0610041E09 gene | 1.82 | 0.00279213 |
| Fundc1 | NM_028058 | FUN14 domain containing 1 | 1.82 | 0.00626899 |
| Bnip2 | NM_001008238 | BCL2/adenovirus E1B 19kDa-interacting protein 1, NIP2 | 1.82 | 0.01067745 |
| Cpeb4 | NM_026252 | cytoplasmic polyadenylation element binding protein 4 | 1.82 | 0.02039929 |
| Mapre1 | NM_007896 | microtubule-associated protein, RP/EB family, member 1 | 1.82 | 0.00726451 |
| Pcm1 | NM_023662 | Pericentriolar material 1 | 1.82 | 0.0191392 |
| Pik3r1 | NM_001024955 | phosphatidylinositol 3-kinase, regulatory subunit, polypeptide 1 (p85 alpha) | 1.82 | 0.00117662 |
| Cast | NM_009817 | calpastatin | 1.83 | 0.00571943 |
| Ddx6 | NM_007841 | DEAD (Asp-Glu-Ala-Asp) box polypeptide 6 | 1.83 | 0.01065922 |
| Fgfr1op2 | NM_026218 | FGFR1 oncogene partner 2 | 1.83 | 0.01615622 |
| Herc5 | NM_025992 | hect domain and RLD 5 | 1.83 | 0.00064199 |
| Hmgcs1 | NM_145942 | 3-hydroxy-3-methylglutaryl-Coenzyme A synthase 1 | 1.83 | 0.0203999 |
| Rap2c | NM_172413 | RAP2C, member of RAS oncogene family | 1.83 | 0.01288078 |
| Sypl | NM_013635 | synaptophysin-like protein | 1.83 | 0.00679224 |
| 9630015D15Rik | NM_181401 | RIKEN cDNA 9630015D15 gene | 1.84 | 0.00723733 |
| 1110001C20Rik | NM_177730 | RIKEN cDNA 1110001C20 gene | 1.84 | 0.00101056 |
| 6130401J04Rik | XM_484835 | RIKEN cDNA 6130401J04 gene | 1.84 | 0.00741181 |
| Sfrs10 | NM_009186 | splicing factor, arginine/serine-rich 10 (transformer 2 homolog, | 1.84 | 0.01364957 |
| Wasl | NM_028459 | Wiskott-Aldrich syndrome-like (human) | 1.84 | 0.00150343 |
| Trim2 | NM_030706 | tripartite motif protein 2 | 1.84 | 0.00387562 |
| Catnal1 | NM_018761 | catenin alpha-like 1 | 1.85 | 0.01377355 |
| Fusip1 | NM_010178 | FUS interacting protein (serine-arginine rich) 1 | 1.85 | 0.00288968 |
| Sh3d1B | NM_011365 | SH3 domain protein 1B | 1.85 | 0.0055675 |
| A630007B06Rik | NM_170757 | RIKEN cDNA A630007B06 gene | 1.85 | 0.00243781 |
| B930006L02Rik | NM_178764 | RIKEN cDNA B930006L02 gene | 1.86 | 0.00541056 |
| Stx6 | NM_021433 | syntaxin 6 | 1.86 | 0.00116142 |
| Sema3c | NM_013657 | sema domain, immunoglobulin domain (Ig), short basic domain, sec | 1.86 | 0.02539057 |
| Jarid1a | XM_359326 | jumonji, AT rich interactive domain 1A (Rbp2 like) | 1.86 | 0.0060163 |
| Btbd7 | NM_172806 | BTB (POZ) domain containing 7 | 1.86 | 0.00492777 |
| 1810013L24Rik | XM_148044 | RIKEN cDNA 1810013L24 gene | 1.86 | 0.0036051 |
| Zbtb20 | NM_019778 | zinc finger and BTB domain containing 20 | 1.87 | 0.00135499 |
| Il13ra1 | NM_133990 | interleukin 13 receptor, alpha 1 | 1.87 | 0.00083911 |
| Apc | NM_007462 | adenomatosis polyposis coli | 1.87 | 0.02970148 |
| Ccm1 | NM_030675 | cerebral cavernous malformations 1 | 1.87 | 0.01695079 |
| Col4a3bp | NM_023420 | procollagen, type IV, alpha 3 (Goodpasture antigen) binding prot | 1.87 | 0.01115037 |
| Mak3 | NM_028108 | Mak3 homolog (S. cerevisiae) | 1.87 | 0.00847124 |
| Mmp19 | NM_021412 | matrix metalloproteinase 19 | 1.87 | 0.01997599 |
| Arl1 | NM_025859 | ADP-ribosylation factor-like 1 | 1.87 | 0.00162835 |
| 6530407C02Rik | NM_133853 | RIKEN cDNA 6530407C02 gene | 1.88 | 0.00533601 |
| Smad7 | NM_008543 | MAD homolog 7 (Drosophila) (Smad7), mRNA | 1.88 | 0.00145137 |
| D9Wsu20e | NM_133718 | DNA segment, Chr 9, Wayne State University 20, expressed | 1.88 | 0.00403625 |
| Rnf134 | NM_027654 | ring finger protein 134 | 1.88 | 0.00670673 |
| Emr4 | NM_139138 | EGF-like module containing, mucin-like, hormone receptor-like se | 1.88 | 0.00070994 |
| Bmi1 | NM_007552 | B lymphoma Mo-MLV insertion region 1 | 1.88 | 0.00591574 |
| Plekha3 | NM_031256 | pleckstrin homology domain-containing, family A (phosphoinositid | 1.89 | 0.00495793 |
| Ggps1 | NM_010282 | geranylgeranyl diphosphate synthase 1 | 1.89 | 0.00277067 |
| Il12b | NM_008352 | interleukin 12b | 1.89 | 0.04353995 |
| Wwp1 | XM_130163 | WW domain containing E3 ubiquitin protein ligase 1 | 1.89 | 0.01074733 |
| A230046K03Rik | XM_193573 | RIKEN cDNA A230046K03 gene | 1.89 | 0.00259294 |
| Mxd1 | NM_010751 | MAX dimerization protein 1 | 1.89 | 0.00348717 |
| Anxa4 | NM_013471 | annexin A4 | 1.89 | 0.00405555 |
| Hrpt2 | NM_145991 | hyperparathyroidism 2 homolog (human) | 1.89 | 0.00576051 |
| B230120H23Rik | NM_023057 | RIKEN cDNA B230120H23 gene | 1.90 | 0.00204685 |
| Cul4a | NM_146207 | cullin 4A | 1.90 | 0.01516488 |
| Fasl | NM_010177 | Fas ligand (TNF superfamily, member 6) | 1.90 | 0.00508764 |
| Icsbp1 | NM_008320 | interferon consensus sequence binding protein 1 | 1.90 | 0.00755809 |
| Tgfbr2 | NM_009371 | transforming growth factor, beta receptor II | 1.90 | 0.00088877 |
| Tlk2 | NM_011903 | Tousled-like kinase 2 (Arabidopsis) | 1.90 | 0.00482623 |
| Usp9x | NM_009481 | ubiquitin specific peptidase 9, X chromosome | 1.90 | 0.00070651 |
| D3Ucla1 | NM_030685 | DNA segment, Chr 3, University of California at Los Angeles 1 | 1.90 | 0.00801724 |
| 1500041J02Rik | NM_026424 | RIKEN cDNA 1500041J02 gene | 1.91 | 0.00401202 |
| 6330441O12Rik | XM_284697 | RIKEN cDNA 6330441O12 gene | 1.91 | 0.00854158 |
| Capn7 | NM_009796 | calpain 7 | 1.91 | 0.01753935 |
| Flrt3 | NM_178382 | fibronectin leucine rich transmembrane protein 3 | 1.91 | 0.0142092 |
| B230219D22Rik | NM_181278 | RIKEN cDNA B230219D22 gene | 1.92 | 0.00640119 |
| Lepr | NM_010704 | leptin receptor | 1.92 | 0.01135667 |
| C920006C10Rik | NM_133766 | RIKEN cDNA C920006C10 gene | 1.92 | 0.01082614 |
| Ppm1b | NM_011151 | protein phosphatase 1B, magnesium dependent, beta isoform | 1.93 | 0.00072865 |
| Cox7c | NM_007749 | cytochrome c oxidase, subunit VIIc | 1.93 | 0.00156272 |
| Arih1 | NM_019927 | ariadne ubiquitin-conjugating enzyme E2 binding protein homolog | 1.93 | 0.00354265 |
| D8Ertd69e | XM_194424 | DNA segment, Chr 8, ERATO Doi 69, expressed | 1.93 | 0.01419258 |
| Dnaja2 | NM_019794 | DnaJ (Hsp40) homolog, subfamily A, member 2 | 1.93 | 0.01207081 |
| Satb1 | NM_009122 | Special AT-rich sequence binding protein 1 | 1.93 | 0.03743118 |
| Csf3r | NM_007782 | colony stimulating factor 3 receptor (granulocyte) | 1.93 | 0.00337734 |
| Hsd17b12 | NM_019657 | hydroxysteroid (17-beta) dehydrogenase 12 | 1.94 | 0.00210385 |
| H3f3a | NM_008210 | H3 histone, family 3A | 1.94 | 0.01397486 |
| MGI:1914262 | NM_133352 | SM-11044 binding protein | 1.94 | 0.00505808 |
| Rnf138 | NM_019706 | ring finger protein 138 | 1.94 | 0.0153214 |
| Stom | NM_013515 | stomatin | 1.94 | 0.00200805 |
| Tm7sf1 | NM_031999 | transmembrane 7 superfamily member 1 | 1.94 | 0.03138958 |
| Ube2v2 | NM_023585 | ubiquitin-conjugating enzyme E2 variant 2 | 1.94 | 0.01315946 |
| Rab27a | NM_023635 | RAB27A, member RAS oncogene family | 1.94 | 0.00477081 |
| Rbbp4 | NM_009030 | retinoblastoma binding protein 4 | 1.95 | 0.00978914 |
| Ssa2 | NM_013835 | Sjogren syndrome antigen A2 | 1.95 | 0.00088603 |
| C430010P07Rik | NM_177645 | RIKEN cDNA C430010P07 gene | 1.95 | 0.00208015 |
| Taok1 | NM_144825 | TAO kinase 1 | 1.95 | 0.00401566 |
| Esco1 | NM_144542 | establishment of cohesion 1 homolog 1 (S. cerevisiae) | 1.95 | 0.00121537 |
| Senp2 | NM_029457 | SUMO/sentrin specific peptidase 2 | 1.95 | 0.0028404 |
| Nfkbiz | NM_030612 | nuclear factor of kappa light polypeptide gene enhancer in B-cells inhibitor, zeta | 1.96 | 0.00111656 |
| 2810485I05Rik | NM_176836 | RIKEN cDNA 2810485I05 gene | 1.96 | 0.00408113 |
| 2700089E24Rik | XM_355822 | RIKEN cDNA 2700089E24 gene | 1.96 | 0.00709094 |
| Ches1 | NM_183186 | checkpoint supressor 1 | 1.97 | 0.00142981 |
| Mtpn | NM_008098 | myotrophin | 1.97 | 0.00516708 |
| Bcl10 | --- | B-cell leukemia/lymphoma 10 | 1.97 | 0.02540293 |
| Chd1 | NM_007690 | chromodomain helicase DNA binding protein 1 | 1.97 | 0.00457735 |
| Ythdf3 | NM_172677 | YTH domain family 3 | 1.97 | 0.00346171 |
| Prkar2a | NM_008924 | protein kinase, cAMP dependent regulatory, type II alpha | 1.98 | 0.00123221 |
| Phr1 | NM_207215 | pam, highwire, rpm 1 | 1.98 | 0.0071168 |
| Map4k3 | XM_128800 | mitogen-activated protein kinase kinase kinase kinase 3 | 1.98 | 0.00216302 |
| Papola | NM_011112 | poly (A) polymerase alpha | 1.98 | 0.00971484 |
| Trpm7 | NM_021450 | Transient receptor potential cation channel, subfamily M, member | 1.98 | 0.00408928 |
| Bmpr1a | NM_009758 | bone morphogenetic protein receptor, type 1A | 1.99 | 0.00228767 |
| Cpsf6 | XM_483955 | cleavage and polyadenylation specific factor 6 | 1.99 | 0.00203336 |
| Mpp6 | NM_019939 | Membrane protein, palmitoylated 6 (MAGUK p55 subfamily member 6) | 1.99 | 0.00203093 |
| Clic5 | NM_172621 | chloride intracellular channel 5 | 2.00 | 0.00254166 |
| D330037H05Rik | XM_128090 | RIKEN cDNA D330037H05 gene | 2.00 | 0.00250355 |
| Kpna3 | NM_008466 | karyopherin (importin) alpha 3 | 2.00 | 0.0085003 |
| Mospd2 | XM_136156 | motile sperm domain containing 2 | 2.00 | 0.00419453 |
| Cct4 | NM_009837 | chaperonin subunit 4 (delta) | 2.00 | 0.00108305 |
| Dstn | NM_019771 | Destrin (Dstn), mRNA | 2.00 | 0.00051566 |
| Kif1b | NM_008441 | kinesin family member 1B | 2.01 | 0.00123668 |
| Map4k5 | NM_024275 | mitogen-activated protein kinase kinase kinase kinase 5 | 2.01 | 0.00801553 |
| Lysmd3 | NM_030257 | LysM, putative peptidoglycan-binding, domain containing 3 | 2.01 | 0.00110996 |
| Ccr7 | NM_007719 | chemokine (C-C motif) receptor 7 | 2.01 | 0.03217454 |
| Npm1 | NM_008722 | nucleophosmin 1 | 2.01 | 0.01254451 |
| Rbpms | NM_019733 | RNA binding protein gene with multiple splicing | 2.01 | 0.0165307 |
| Tceb1 | NM_026456 | transcription elongation factor B (SIII), polypeptide 1 | 2.01 | 0.01345583 |
| Ube1c | NM_011666 | ubiquitin-activating enzyme E1C | 2.01 | 0.00498908 |
| Tbc1d15 | NM_025706 | TBC1 domain family, member 15 | 2.01 | 0.00232162 |
| Dnm1l | NM_001025947 | dynamin 1-like | 2.01 | 0.00091862 |
| Cftr | NM_021050 | cystic fibrosis transmembrane conductance regulator homolog | 2.01 | 0.00315599 |
| D13Wsu64e | NM_172585 | DNA segment, Chr 13, Wayne State University 64, expressed, mRNA | 2.01 | 0.00072081 |
| Plxnc1 | NM_018797 | plexin C1 | 2.01 | 0.00878436 |
| Sh3rf1 | NM_021506 | SH3 domain containing ring finger 1 | 2.01 | 0.00128126 |
| Trove2 | NM_013835 | TROVE domain family, member 2 | 2.01 | 0.00088603 |
| Ap3b1 | NM_009680 | adaptor-related protein complex 3, beta 1 subunit | 2.02 | 0.00106507 |
| Casp3 | NM_009810 | caspase 3, apoptosis related cysteine protease | 2.02 | 0.01634576 |
| Ptprk | NM_008983 | protein tyrosine phosphatase, receptor type, K | 2.02 | 0.01010987 |
| Phca | NM_025408 | phytoceramidase, alkaline | 2.02 | 0.00142702 |
| Ptp4a1 | NM_011200 | protein tyrosine phosphatase 4a1 | 2.03 | 0.00234644 |
| Actl6a | NM_019673 | actin-like 6A | 2.03 | 0.00051113 |
| Fgg | NM_133862 | fibrinogen, gamma polypeptide | 2.03 | 0.01440504 |
| Il1r2 | NM_010555 | interleukin 1 receptor, type II | 2.03 | 0.02901957 |
| Ptpn12 | NM_011203 | Protein tyrosine phosphatase, non-receptor type 12 | 2.03 | 0.00218071 |
| Peli1 | NM_023324 | pellino 1 | 2.03 | 0.00121526 |
| Wac | NM_153085 | WW domain containing adaptor with coiled-coil | 2.03 | 0.00112138 |
| 1110001M19Rik | NM_001024205 | RIKEN cDNA 1110001M19 gene | 2.03 | 0.0008677 |
| E030004N02Rik |  | RIKEN cDNA E030004N02 gene | 2.03 | 0.00461501 |
| 2310036D22Rik | NM_027992 | RIKEN cDNA 2310036D22 gene | 2.04 | 0.02046244 |
| 5730538E15Rik | NM_173443 | RIKEN cDNA 5730538E15 gene | 2.04 | 0.00107794 |
| Cdc73 | NM_145991 | Vcell division cycle 73, Paf1/RNA polymerase II complex component | 2.04 | 0.00064451 |
| Ank3 | NM_009670 | ankyrin 3, epithelial | 2.05 | 0.00073181 |
| Ppp1cb | NM_172707 | protein phosphatase 1, catalytic subunit, beta isoform | 2.05 | 0.00697975 |
| Arfgef1 | XM_129376 | ADP-ribosylation factor guanine nucleotide-exchange factor 1 | 2.05 | 0.00051549 |
| Nbn | NM_013752 | nibrin | 2.05 | 0.00090376 |
| 6330549H03Rik | NM_031391 | RIKEN cDNA 6330549H03 gene | 2.05 | 0.03196966 |
| Mgea5 | NM_023799 | meningioma expressed antigen 5 (hyaluronidase) | 2.05 | 0.01706494 |
| Rpl5 | NM_016980 | ribosomal protein L5 | 2.05 | 0.00184172 |
| Ccr1 | NM_009912 | chemokine (C-C motif) receptor 1 | 2.06 | 0.02824321 |
| Rasa1 | NM_145452 | RAS p21 protein activator 1 | 2.06 | 0.00071551 |
| Vcpip1 | NM_173443 | valosin containing protein (p97)/p47 complex interacting protein 1 | 2.06 | 0.00107794 |
| Csnk1g3 | NM_152809 | Casein kinase 1, gamma 3 (Csnk1g3), mRNA | 2.06 | 0.00064855 |
| Ms4a7 | NM_001025610 | membrane-spanning 4-domains, subfamily A, member 7 | 2.07 | 0.00059926 |
| C230027N18Rik |  | RIKEN cDNA C230027N18 gene | 2.07 | 0.00315658 |
| Gnai1 | NM_010305 | guanine nucleotide binding protein, alpha inhibiting 1 | 2.07 | 0.00054973 |
| Il1rn | NM_031167 | interleukin 1 receptor antagonist | 2.07 | 0.01574673 |
| Il2rb | NM_008368 | Interleukin 2 receptor, beta chain | 2.07 | 0.01612455 |
| Ms4a1 | NM_007641 | membrane-spanning 4-domains, subfamily A, member 1 | 2.07 | 0.04195869 |
| Psme4 | NM_134013 | proteasome (prosome, macropain) activator subunit 4 | 2.07 | 0.00863709 |
| Ddx26 | NM_008715 | DEAD/H (Asp-Glu-Ala-Asp/His) box polypeptide 26 | 2.07 | 0.00073138 |
| Xpo4 | NM_020506 | exportin 4 | 2.07 | 0.00048264 |
| Csnk1a1 | NM_146087 | casein kinase 1, alpha 1 | 2.08 | 0.00076158 |
| Kif5b | NM_008448 | kinesin family member 5B | 2.08 | 0.00576622 |
| Pde7a | NM_008802 | phosphodiesterase 7A | 2.08 | 0.00481629 |
| Vps54 | NM_139061 | vacuolar protein sorting 54 (yeast) | 2.08 | 0.0010489 |
| Slmap | NM_032008 | sarcolemma associated protein | 2.08 | 0.00067405 |
| Pum2 | NM_030723 | pumilio 2 (Drosophila) | 2.08 | 0.00901484 |
| Marcksl1 | NM_010807 | MARCKS-like 1 | 2.08 | 0.00079403 |
| Fbxl11 | NM_001001984 | MKIAA1004 protein | 2.09 | 0.00114313 |
| Psmd14 | NM_021526 | Proteasome (prosome, macropain) 26S subunit, non-ATPase, 14, mRNA | 2.09 | 0.00132064 |
| Acbd3 | NM_133225 | acyl-Coenzyme A binding domain containing 3 | 2.09 | 0.00293953 |
| Bzw1 | NM_025824 | basic leucine zipper and W2 domains 1 | 2.09 | 0.01647436 |
| Pbef1 | NM_021524 | pre-B-cell colony-enhancing factor 1 | 2.09 | 0.00050917 |
| Atp6v0a1 | NM_016920 | ATPase, H+ transporting, lysosomal V0 subunit a isoform 1 | 2.09 | 0.00373775 |
| Lpin2 | NM_022882 | Lipin 2 | 2.09 | 0.00152199 |
| Heca | XM_354533 | headcase homolog (Drosophila) | 2.09 | 0.00037659 |
| Dleu2 | --- | deleted in lymphocytic leukemia, 2 | 2.10 | 0.00076822 |
| Ppp2r5e | NM_012024 | MKIAA4006 protein | 2.10 | 0.00104258 |
| 1110030H10Rik | NM_030257 | RIKEN cDNA 1110030H10 gene | 2.10 | 0.00110996 |
| D10627 | --- | cDNA sequence D10627 | 2.10 | 0.00125105 |
| Smurf2 | NM_025481 | SMAD specific E3 ubiquitin protein ligase 2 | 2.10 | 0.00045461 |
| 5830435K17Rik | NM_175318 | RIKEN cDNA 5830435K17 gene | 2.10 | 0.00286862 |
| Abcb7 | XM_356348 | ATP-binding cassette, sub-family B (MDR/TAP), member 7 | 2.10 | 0.00172191 |
| Ppp3cb | NM_008914 | protein phosphatase 3, catalytic subunit, beta isoform | 2.10 | 0.02542642 |
| Smarca5 | NM_053124 | SWI/SNF related, matrix associated, actin dependent regulator of | 2.10 | 0.00781871 |
| Tnfrsf12a | NM_013749 | tumor necrosis factor receptor superfamily, member 12a | 2.10 | 0.02266441 |
| Ttc1 | NM_133795 | tetratricopeptide repeat domain 1 | 2.10 | 0.00074826 |
| C130068B02Rik | --- | RIKEN cDNA C130068B02 gene | 2.10 | 0.00044597 |
| B430201A12Rik | XM_283903 | RIKEN cDNA B430201A12 gene | 2.10 | 0.00574583 |
| Vti1a | NM_016862 | vesicle transport through interaction with t-SNAREs homolog 1A (yeast) | 2.10 | 0.00105573 |
| 6330407A03Rik | --- | RIKEN cDNA 6330407A03 gene | 2.10 | 0.00063051 |
| Chm | NM_018818 | choroidermia | 2.11 | 0.00314366 |
| Cpeb2 | NM_175937 | cytoplasmic polyadenylation element binding protein 2 | 2.11 | 0.02799183 |
| Cyr61 | NM_010516 | cysteine rich protein 61 | 2.11 | 0.01074006 |
| Phip | XM_358384 | pleckstrin homology domain interacting protein | 2.11 | 0.01535576 |
| Slc12a2 | NM_009194 | solute carrier family 12, member 2 | 2.11 | 0.0003453 |
| Herc1 | XM_358383 | Hect (homologous to the E6-AP (UBE3A) carboxyl terminus) domain | 2.12 | 0.00374803 |
| Mtmr6 | NM_144843 | myotubularin related protein 6 | 2.12 | 0.00705044 |
| Eif4a1 | NM_144958 | eukaryotic translation initiation factor 4A1 | 2.12 | 0.00869718 |
| Ybx1 | NM_011732 | Y box protein 1 | 2.13 | 0.00111454 |
| Slit2 | NM_178804 | Slit homolog 2 (Drosophila) | 2.13 | 0.00764408 |
| Atp2a2 | NM_009722 | ATPase, Ca++ transporting, cardiac muscle, slow twitch 2 | 2.13 | 0.00107274 |
| Fgfr2 | NM_010207 | fibroblast growth factor receptor 2 | 2.13 | 0.01499451 |
| Jak2 | NM_008413 | Janus kinase 2 | 2.13 | 0.01000026 |
| Ankrd12 | NM_001025572 | Ankyrin repeat domain 12 | 2.13 | 0.00060617 |
| Yme1l1 | NM_013771 | YME1-like 1 (S. cerevisiae) | 2.13 | 0.00857455 |
| 2610101N10Rik | NM_026476 | RIKEN cDNA 2610101N10 gene | 2.14 | 0.00177805 |
| 2310035C23Rik | NM_173187 | RIKEN cDNA 2310035C23 gene | 2.14 | 0.00463953 |
| Foxp1 | NM_053202 | Forkhead box P1 (Foxp1), mRNA | 2.14 | 0.00095084 |
| Igf1 | NM_010512 | Insulin-like growth factor 1, mRNA | 2.14 | 0.00276698 |
| D1Ertd646e | --- | DNA segment, Chr 1, ERATO Doi 646, expressed | 2.14 | 0.00125608 |
| Sirt1 | NM_019812 | sirtuin 1 | 2.14 | 0.0010243 |
| Add3 | NM_013758 | adducin 3 (gamma) | 2.15 | 0.00700122 |
| Snx10 | NM_028035 | Sorting nexin 10 (Snx10), mRNA | 2.15 | 0.00068779 |
| Stag1 | NM_009282 | stromal antigen 1 | 2.15 | 0.00345228 |
| Marco | NM_010766 | macrophage receptor with collagenous structure | 2.15 | 0.00510265 |
| Mpp7 | XM_128966 | membrane protein, palmitoylated 7 (MAGUK p55 subfamily member 7) | 2.15 | 0.00842959 |
| D10Wsu52e | NM_145422 | DNA segment, Chr 10, Wayne State University 52, expressed. | 2.15 | 0.00106705 |
| Yy1 | NM_009537 | YY1 transcription factor (Yy1), mRNA | 2.15 | 0.00076662 |
| Acvrinp1 | NM_015823 | activin receptor interacting protein 1 | 2.16 | 0.00301063 |
| Bach2 | NM_007521 | BTB and CNC homology 2 (Bach2), mRNA | 2.16 | 0.00038114 |
| Ccl6 | NM_009139 | chemokine (C-C motif) ligand 6 | 2.16 | 0.00253498 |
| Cldn1 | NM_016674 | claudin 1 | 2.17 | 0.00189683 |
| Tlk1 | NM_172664 | MKIAA0137 protein | 2.17 | 0.00050973 |
| Dusp16 | NM_130447 | dual specificity phosphatase 16 | 2.18 | 0.00039761 |
| Icos | NM_017480 | inducible T-cell co-stimulator | 2.18 | 0.00038505 |
| Cd300lf | NM_145634 | CD300 antigen like family member F | 2.18 | 0.00078602 |
| Lactb2 | NM_145381 | lactamase, beta 2 | 2.18 | 0.02820985 |
| Csf2rb1 | NM_007780 | colony stimulating factor 2 receptor, beta 1 | 2.18 | 0.00446654 |
| 1700019B16Rik | NM_028829 | Progestin and adipoQ receptor family member VIII (Paqr8) | 2.19 | 0.00139985 |
| Cul3 | NM_016716 | cullin 3 | 2.19 | 0.01595749 |
| D16Ertd472e | NM_025967 | DNA segment, Chr 16, ERATO Doi 472, expressed | 2.19 | 0.02180607 |
| Rod1 | NM_144904 | ROD1 regulator of differentiation 1 (S. pombe) | 2.19 | 0.00774358 |
| Son | NM_019973 | Son cell proliferation protein | 2.20 | 0.00135875 |
| Trpm6 | NM_153417 | transient receptor potential cation channel, subfamily M, member 6 | 2.20 | 0.0005624 |
| Pcqap | NM_033609 | positive cofactor 2, multiprotein complex, glutamine/Q-rich-associated protein | 2.20 | 0.00051893 |
| Cdk6 | NM_009873 | Cyclin-dependent kinase 6 (Cdk6), mRNA | 2.21 | 0.00046527 |
| Pdcd6ip | NM_011052 | programmed cell death 6 interacting protein | 2.21 | 0.00063151 |
| MGC79224 | NM_010658 | basic domain/leucine zipper transcription factor | 2.21 | 0.00191042 |
| Pcf11 | NM_029078 | cleavage and polyadenylation factor subunit homolog (S. cerevisiae) | 2.21 | 0.00118795 |
| 3830421F13Rik | NM_027226 | RIKEN cDNA 3830421F13 gene | 2.21 | 0.0003752 |
| AA407452 | XM_145140 | EST AA407452 | 2.21 | 0.00240233 |
| Timp2 | NM_011594 | tissue inhibitor of metalloproteinase 2 | 2.22 | 0.0011072 |
| Ifnar1 | NM_010508 | Interferon (alpha and beta) receptor 1 | 2.22 | 0.01193009 |
| Runx1 | NM_009821 | runt related transcription factor 1 | 2.22 | 0.00323827 |
| AV216087 | NM_144804 | expressed sequence AV216087 | 2.23 | 0.00085204 |
| Ubxd2 | NM_026390 | UBX domain containing 2 | 2.23 | 0.00124436 |
| 6330415F13Rik | NM_027533 | RIKEN cDNA 6330415F13 gene | 2.23 | 0.00178898 |
| Sap18 | NM_009119 | Sin3-associated polypeptide 18 | 2.23 | 0.00307999 |
| Stat4 | NM_011487 | signal transducer and activator of transcription 4 | 2.23 | 0.02942509 |
| 2810051F02Rik | NM_028330 | RIKEN cDNA 2810051F02 gene | 2.23 | 0.00587913 |
| Spred1 | NM_033524 | sprouty protein with EVH-1 domain 1, related sequence | 2.23 | 0.00497622 |
| Slc35a3 | NM_144902 | solute carrier family 35 (UDP-N-acetylglucosamine (UDP-GlcNAc) t | 2.23 | 0.00801903 |
| Za20d2 | NM_009551 | zinc finger, A20 domain containing 2 | 2.23 | 0.00071294 |
| Hip2 | NM_016786 | huntingtin interacting protein 2 | 2.24 | 0.00565344 |
| C1qb | NM_009777 | complement component 1, q subcomponent, beta polypeptide | 2.24 | 0.0051649 |
| Rbbp6 | NM_011247 | Retinoblastoma binding protein 6 (Rbbp6), transcript variant 1, mRNA | 2.25 | 0.00128282 |
| Rp2h | NM_133669 | retinitis pigmentosa 2 homolog (human) | 2.25 | 0.00764086 |
| 2600011C06Rik | NM_025930 | RIKEN cDNA 2600011C06 gene | 2.25 | 0.02541172 |
| 4832420A03Rik |  | RIKEN cDNA 4832420A03 gene | 2.25 | 0.01106442 |
| Ap3m1 | NM_018829 | adaptor-related protein complex 3, mu 1 subunit | 2.26 | 0.00118703 |
| A530068K01 |  | hypothetical protein A530068K01 | 2.26 | 0.00696366 |
| Dlgh1 | NM_007862 | Discs, large homolog 1 (Drosophila) | 2.26 | 0.00660786 |
| Npnt | NM_033525 | Nephronectin | 2.26 | 0.00184083 |
| Tiparp | NM_178892 | TCDD-inducible poly(ADP-ribose) polymerase | 2.26 | 0.00107264 |
| 4432406C05Rik | NM_025708 | RIKEN cDNA 4432406C05 gene | 2.26 | 0.00028974 |
| 2810407C02Rik | XM_283848 | RIKEN cDNA 2810407C02 gene | 2.27 | 0.02397908 |
| 5033406L14Rik | NM_029492 | RIKEN cDNA 5033406L14 gene | 2.27 | 0.01151344 |
| Lims1 | NM_026148 | LIM and senescent cell antigen-like domains 1 | 2.27 | 0.00869915 |
| E030026I10Rik | NM_175553 | RIKEN cDNA E030026I10 gene | 2.28 | 0.00300176 |
| Pten | NM_008960 | phosphatase and tensin homolog | 2.28 | 0.00584796 |
| Bcl2l11 | NM_009754 | BCL2-like 11 (apoptosis facilitator) | 2.28 | 0.00231144 |
| Phf20l1 | NM_178718 | PHD finger protein 20-like 1 | 2.28 | 0.02411645 |
| Suz12 | NM_199196 | suppressor of zeste 12 homolog (Drosophila) | 2.28 | 0.02872387 |
| Ube2d3 | NM_025356 | ubiquitin-conjugating enzyme E2D 3 (UBC4/5 homolog, yeast) | 2.29 | 0.0097512 |
| LOC233467 | NM_011723 | hypothetical gene | 2.29 | 0.02280939 |
| Ptpns1 | NM_007547 | protein tyrosine phosphatase, non-receptor type substrate 1 | 2.29 | 0.00190686 |
| 3110001A13Rik | NM_025626 | RIKEN cDNA 3110001A13 gene, mRNA | 2.29 | 0.00138099 |
| Lin7c | NM_011699 | lin 7 homolog c (C. elegans) | 2.30 | 0.00161785 |
| A130001D14Rik | --- | RIKEN cDNA A130001D14 gene | 2.30 | 0.00052523 |
| Jarid1c | NM_013668 | jumonji, AT rich interactive domain 1C (Rbp2 like) | 2.30 | 0.00027302 |
| Rps6 | NM_009096 | Ribosomal protein S6, mRNA | 2.31 | 0.00115118 |
| Capza2 | NM_007604 | capping protein (actin filament) muscle Z-line, alpha 2 | 2.31 | 0.00515121 |
| Api5 | NM_007466 | Apoptosis inhibitor 5, mRNA | 2.31 | 0.00026435 |
| Rb1cc1 | NM_009826 | RB1-inducible coiled-coil 1 | 2.31 | 0.01625003 |
| Sec24b | NM_207209 | SEC24 related gene family, member B (S. cerevisiae) | 2.31 | 0.00253702 |
| Cd207 | NM_144943 | CD 207 antigen | 2.31 | 0.0002527 |
| Il1b | NM_008361 | interleukin 1 beta | 2.31 | 0.00137941 |
| Ing3 | NM_023626 | inhibitor of growth family, member 3 | 2.31 | 0.00022811 |
| Clec4e | NM_019948 | C-type lectin domain family 4, member e | 2.32 | 0.02030457 |
| 2810429K17Rik | NM_029847 | RIKEN cDNA 2810429K17 gene | 2.32 | 0.00051935 |
| Tnfsf6 | NM_010177 | tumor necrosis factor (ligand) superfamily, member 6 | 2.33 | 0.00508764 |
| Osbpl8 | NM_001003717 | Oxysterol binding protein-like 8 | 2.33 | 0.00227605 |
| Pscdbp | NM_139200 | pleckstrin homology, Sec7 and coiled-coil domains, binding prote | 2.33 | 0.00888853 |
| Styx | NM_019637 | phosphoserine/threonine/tyrosine interaction protein | 2.33 | 0.0010564 |
| Ehf | NM_007914 | ets homologous factor | 2.33 | 0.00066531 |
| Pkn2 | NM_178654 | protein kinase N2 | 2.34 | 0.00850156 |
| Birc6 | NM_007566 | baculoviral IAP repeat-containing 6 | 2.34 | 0.00032656 |
| D19Ertd703e | NM_029456 | DNA segment, Chr 19, ERATO Doi 703, expressed | 2.34 | 0.01098722 |
| Ide | NM_031156 | insulin degrading enzyme | 2.34 | 0.00621566 |
| Ube2d2 | NM_019912 | Ubiquitin-conjugating enzyme E2D 2 | 2.34 | 0.004843 |
| Mtmr1 | NM_016985 | myotubularin related protein 1 | 2.34 | 0.00723251 |
| Rhoe | NM_028810 | ras homolog gene family, member E | 2.34 | 0.00244412 |
| AU023006 | --- | expressed sequence AU023006 | 2.35 | 0.00048305 |
| AI314180 | NM_172381 | expressed sequence AI314180 | 2.35 | 0.0012518 |
| Cdc27 | NM_145436 | Cell division cycle 27 homolog (S. cerevisiae) | 2.35 | 0.00415429 |
| Ggnbp2 | NM_153144 | Gametogenetin binding protein 2 | 2.35 | 0.00036002 |
| Cd86 | NM_019388 | CD86 antigen | 2.36 | 0.00191646 |
| Rfwd2 | NM_011931 | Constitutive photomorphogenic protein (Cop1) | 2.36 | 0.00033541 |
| Thrap3 | NM_146153 | thyroid hormone receptor associated protein 3 | 2.36 | 0.01497669 |
| Gcl | NM_011818 | germ cell-less homolog (Drosophila) | 2.36 | 0.0010104 |
| Pftk1 | NM_011074 | PFTAIRE protein kinase 1 (Pftk1), mRNA | 2.36 | 0.00133518 |
| Rora | NM_013646 | RAR-related orphan receptor alpha, mRNA | 2.36 | 0.00055788 |
| Ddef1 | NM_010026 | RIKEN cDNA 1700010G06 gene | 2.37 | 0.00839179 |
| 2810474O19Rik | NM_026054 | RIKEN cDNA 2810474O19 gene | 2.37 | 0.00105461 |
| Crebbp | XM_148699 | CREB binding protein | 2.37 | 0.00114706 |
| Man1a | NM_008548 | Mannosidase 1, alpha | 2.38 | 0.01570362 |
| D3Jfr1 | NM_144901 | DNA segment, Chr 3, MJeffers 1 | 2.38 | 0.00060174 |
| Rpl5 | NM_016980 | ribosomal protein L5 | 2.38 | 0.00070128 |
| Fnbp3 | NM_018785 | Formin binding protein 3 | 2.38 | 0.00816453 |
| Ramp2 | NM_019444 | receptor (calcitonin) activity modifying protein 2 | 2.38 | 0.00771886 |
| Sod1 | NM_019706 | Superoxide dismutase 1, soluble | 2.38 | 0.0038696 |
| Cnot7 | NM_011135 | CCR4-NOT transcription complex, subunit 7 | 2.39 | 0.00890705 |
| Rnf2 | NM_011277 | ring finger protein 2 | 2.40 | 0.00079537 |
| Clock | NM_007715 | circadian locomoter output cycles kaput | 2.40 | 0.00069411 |
| Sumo1 | NM_009460 | SMT3 suppressor of mif two 3 homolog 1 (yeast) (Sumo1), mRNA | 2.41 | 0.00092638 |
| Gas2l3 | XM_137276 | growth arrest-specific 2 like 3 | 2.41 | 0.00078037 |
| 9630009C16 | --- | hypothetical protein 9630009C16 | 2.41 | 0.00117318 |
| Matr3 | NM_010771 | matrin 3 | 2.41 | 0.01057844 |
| Qk | NM_021881 | Quaking (Qk), mRNA | 2.41 | 0.00019665 |
| P4ha1 | NM_011030 | procollagen-proline, 2-oxoglutarate 4-dioxygenase, alpha 1 polypeptide | 2.41 | 0.00017895 |
| 2500001H09Rik | NM_029078 | RIKEN cDNA 2500001H09 gene | 2.41 | 0.00163652 |
| 4632427E13Rik | --- | RIKEN cDNA 4632427E13 gene | 2.42 | 0.00037297 |
| Mbnl3 | NM_134163 | muscleblind-like 3 (Drosophila) | 2.43 | 0.01168467 |
| Magi2 | NM_015823 | membrane associated guanylate kinase, WW and PDZ domain containing 2 | 2.44 | 0.00019733 |
| Csf2ra | NM_009970 | colony stimulating factor 2 receptor, alpha | 2.44 | 0.0078415 |
| 1110003F05Rik | --- | RIKEN cDNA 1110003F05 gene | 2.45 | 0.00042794 |
| Rgs2 | NM_009061 | regulator of G-protein signaling 2 | 2.45 | 0.01261942 |
| 9430012M22Rik | --- | RIKEN cDNA 9430012M22 gene | 2.45 | 0.000386 |
| Rai14 | NM_030690 | retinoic acid induced 14 | 2.46 | 0.00313957 |
| Crk7 | NM_026952 | CDC2-related kinase 7 | 2.46 | 0.00315505 |
| BC049806 | NM_172513 | CDNA sequence BC049806 | 2.46 | 0.00469213 |
| Mef2a | NM_013597 | myocyte enhancer factor 2A | 2.46 | 0.0066551 |
| 1700081L11Rik | XM_109700 | RIKEN cDNA D030002E05 gene | 2.46 | 0.02267478 |
| Eif5 | NM_173363 | eukaryotic translation initiation factor 5 | 2.46 | 0.00591114 |
| Dmxl1 | XM_283496 | Dmx-like 1 | 2.46 | 0.00041892 |
| Sri | NM_025618 | sorcin | 2.46 | 0.00034597 |
| Tcf12 | NM_011544 | transcription factor 12 | 2.47 | 0.00158985 |
| Fyttd1 | NM_027226 | forty-two-three domain containing 1 | 2.48 | 0.0003752 |
| C730024G19Rik | XM_132975 | hypothetical protein XP_132975 | 2.48 | 0.0014136 |
| D18Ertd232e | --- | DNA segment, Chr 18, ERATO Doi 232, expressed | 2.48 | 0.00021786 |
| 5832424M12Rik | NM_172591 | RIKEN cDNA 5832424M12 gene | 2.48 | 0.00219039 |
| B630005N14Rik | NM_175312 | RIKEN cDNA B630005N14 gene | 2.48 | 0.00655152 |
| C030038J10Rik | NM_183183 | RIKEN cDNA C030038J10 gene | 2.48 | 0.00591821 |
| 4833414E09Rik | --- | Mus musculus, clone IMAGE:5346272, mRNA | 2.49 | 0.00107046 |
| C1galt1 | NM_052993 | core 1 UDP-galactose:N-acetylgalactosamine-alpha-R beta 1,3-gala | 2.49 | 0.00485284 |
| Rock1 | NM_009071 | Rho-associated coiled-coil forming kinase 1 | 2.49 | 0.03646487 |
| Rgs4 | NM_009062 | regulator of G-protein signaling 4 | 2.49 | 0.00071464 |
| Mpp5 | NM_019579 | membrane protein, palmitoylated 5 (MAGUK p55 subfamily member 5) | 2.50 | 0.0055829 |
| Utx | NM_009483 | Ubiquitously transcribed tetratricopeptide repeat gene, X chromo | 2.50 | 0.00974392 |
| Tmod3 | NM_016963 | Tropomodulin 3 (Tmod3), mRNA | 2.51 | 0.0005126 |
| Mnab | XM_130233 | Membrane associated DNA binding protein | 2.51 | 0.00226547 |
| Sesn3 | NM_030261 | Sestrin 3 | 2.51 | 0.00061083 |
| Ugcg | NM_011673 | UDP-glucose ceramide glucosyltransferase | 2.51 | 0.01875353 |
| Abi1 | NM_007380 | abl-interactor 1 | 2.52 | 0.00432183 |
| Mobk1b | NM_145571 | MOB1, Mps One Binder kinase activator-like 1B (yeast) | 2.53 | 0.0019384 |
| Rnf10 | NM_016698 | Ring finger protein 10, mRNA | 2.53 | 0.00048598 |
| Nrip1 | NM_173440 | nuclear receptor interacting protein 1 | 2.53 | 0.00018529 |
| AI120166 | --- | expressed sequence AI120166 | 2.54 | 0.00076292 |
| Itgb6 | NM_021359 | integrin beta 6 | 2.54 | 0.00025339 |
| Stch | NM_030201 | stress 70 protein chaperone, microsome-associated, human homolog | 2.55 | 0.00051082 |
| Tcea1 | NM_011541 | transcription elongation factor A (SII) 1 | 2.55 | 0.00821177 |
| 8030497I03Rik | --- | RIKEN cDNA 8030497I03 gene | 2.55 | 0.00106159 |
| Dyrk1a | NM_007890 | Dual-specificity tyrosine-(Y)-phosphorylation regulated kinase 1a | 2.56 | 0.00051217 |
| Tmed7 | XM_128959 | transmembrane emp24 protein transport domain containing 7 | 2.56 | 0.00097534 |
| Sh3bgrl | NM_019989 | SH3-binding domain glutamic acid-rich protein like | 2.56 | 0.00099722 |
| Prkca | NM_011101 | Protein kinase C, alpha (Prkca), mRNA | 2.57 | 0.00025849 |
| BC016423 | XM_622100 | cDNA sequence BC016423 | 2.57 | 0.00020124 |
| Etv5 | NM_023794 | ets variant gene 5 | 2.58 | 0.00197089 |
| Cflar | NM_009805 | CASP8 and FADD-like apoptosis regulator | 2.58 | 0.000233 |
| Slc4a7 | XM_147798 | Solute carrier family 4, sodium bicarbonate cotransporter, membe | 2.58 | 0.01254342 |
| Dock7 | NM_026082 | dedicator of cytokinesis 7 | 2.58 | 0.00086934 |
| Golph3 | NM_025673 | golgi phosphoprotein 3 | 2.59 | 0.00336623 |
| Gnaq | NM_008139 | Phosphatidylethanolamine binding protein | 2.59 | 0.0000815 |
| Hmgcs2 | NM_008256 | 3-hydroxy-3-methylglutaryl-Coenzyme A synthase 2 | 2.59 | 0.00073477 |
| Pum1 | NM_030722 | Pumilio 1 (Drosophila) (Pum1), mRNA | 2.59 | 0.00066951 |
| Polr3b | NM_027423 | Polymerase (RNA) III (DNA directed) polypeptide B (Polr3b), mRNA | 2.59 | 0.0007658 |
| 5730403B10Rik | NM_025670 | RIKEN cDNA 5730403B10 gene, mRNA | 2.61 | 0.00024391 |
| B930093H17Rik | --- | RIKEN cDNA B930093H17 gene | 2.61 | 0.00012929 |
| Scoc | NM_019708 | short coiled-coil protein | 2.62 | 0.00708929 |
| Pbx1 | NM_008783 | Pre B-cell leukemia transcription factor 1, mRNA | 2.62 | 0.00138168 |
| C330012H03Rik | NM_183029 | Insulin-like growth factor 2 mRNA-binding protein 2 (Imp2) | 2.63 | 0.00027088 |
| AI451896 | --- | expressed sequence AI451896 | 2.63 | 0.00036002 |
| Oazin | NM_018745 | ornithine decarboxylase antizyme inhibitor | 2.63 | 0.00179449 |
| Ppp1r10 | NM_175934 | protein phosphatase 1, regulatory subunit 10 | 2.64 | 0.00014152 |
| Ppp1r12a | XM_137239 | protein phosphatase 1, regulatory (inhibitor) subunit 12A | 2.64 | 0.00034407 |
| Snap23 | NM_009222 | synaptosomal-associated protein 23 | 2.65 | 0.00157678 |
| Ext1 | NM_010162 | Exostoses (multiple) 1, mRNA | 2.65 | 0.0001828 |
| Dcamkl1 | NM_019978 | double cortin and calcium/calmodulin-dependent protein kinase-like 1 | 2.65 | 0.00142886 |
| Stk3 | NM_019635 | serine/threonine kinase 3 (Ste20, yeast homolog) | 2.66 | 0.01542827 |
| 3110050K21Rik | NM_026083 | RIKEN cDNA 3110050K21 gene, mRNA | 2.66 | 0.00024235 |
| Ero1l | NM_015774 | ERO1-like (S. cerevisiae) | 2.67 | 0.00790808 |
| Fcgr2b | NM_010187 | Fc receptor, IgG, low affinity IIb | 2.67 | 0.01273618 |
| Nup98 | --- | PREDICTED: Mus musculus nucleoporin 98 (Nup98), mRNA | 2.67 | 0.00055699 |
| Snx5 | NM_024225 | sorting nexin 5 | 2.67 | 0.00128008 |
| Rgmb | NM_178615 | RGM domain family, member B | 2.68 | 0.00059491 |
| 4921505C17Rik | NM_030168 | RIKEN cDNA 4921505C17 gene | 2.68 | 0.00024191 |
| Ube3a | NM_001033962 | ubiquitin protein ligase E3A | 2.70 | 0.0006259 |
| E230008O15Rik | --- | RIKEN cDNA E230008O15 gene | 2.70 | 0.00048368 |
| B2m | NM_009735 | Beta-2 microglobulin | 2.71 | 0.00465054 |
| Tmed5 | NM_028876 | transmembrane emp24 protein transport domain containing 5 | 2.72 | 0.00034216 |
| Tm6sf1 | NM_145375 | transmembrane 6 superfamily member 1 | 2.73 | 0.01093196 |
| Stag2 | NM_021465 | stromal antigen 2 | 2.73 | 0.0084176 |
| Nfib | NM_008687 | nuclear factor I/B | 2.73 | 0.00025913 |
| Tpp2 | NM_009418 | tripeptidyl peptidase II | 2.74 | 0.00213603 |
| A730024A03Rik | NM_173753 | RIKEN cDNA A730024A03 gene | 2.74 | 0.00091055 |
| Tgfbr1 | NM_009370 | transforming growth factor, beta receptor I | 2.74 | 0.00109797 |
| 2010106G01Rik | NM_023220 | RIKEN cDNA 2010106G01 gene | 2.75 | 0.00668303 |
| Fbxl3 | NM_015822 | F-box and leucine-rich repeat protein 3 | 2.75 | 0.0017807 |
| Usp9x | NM_009481 | ubiquitin specific protease 9, X chromosome | 2.75 | 0.00182602 |
| 1110014D18Rik | NM_026746 | RIKEN cDNA 1110014D18 gene (1110014D18Rik), mRNA | 2.75 | 0.0007762 |
| 4732464A07Rik | --- | RIKEN cDNA 4732464A07 gene | 2.75 | 0.00106958 |
| Cd53 | NM_007651 | CD53 antigen, mRNA | 2.75 | 0.00012116 |
| 4930553M18Rik | NM_026541 | RIKEN cDNA 4930553M18 gene | 2.78 | 0.00037921 |
| Crem | NM_013498 | cAMP responsive element modulator | 2.78 | 0.00619621 |
| Hdlbp | NM_133808 | high density lipoprotein (HDL) binding protein | 2.79 | 0.00087286 |
| A430106J12Rik | NM_176841 | RIKEN cDNA A430106J12 gene | 2.79 | 0.0103511 |
| Myo1d | NM_177390 | Myosin ID (Myo1d), mRNA | 2.79 | 0.0000958 |
| 2310050N11Rik | XM_193956 | RIKEN cDNA 2310050N11 gene | 2.81 | 0.00183261 |
| Ipo7 | NM_181517 | importin 7 | 2.81 | 0.00014648 |
| Csde1 | NM_144901 | cold shock domain containing E1, RNA binding | 2.81 | 0.00060174 |
| Etnk1 | XM_284250 | ethanolamine kinase 1 | 2.82 | 0.00043736 |
| 1500011J06Rik | NM_001005523 | RIKEN cDNA 1500011J06 gene | 2.82 | 0.00748882 |
| 4930523C07Rik |  | RIKEN cDNA 4930523C07 gene | 2.82 | 0.00076815 |
| Tcte1l | NM_025975 | t-complex-associated-testis-expressed 1-like | 2.82 | 0.01726539 |
| Traf1 | NM_009421 | Tnf receptor-associated factor 1 | 2.82 | 0.00103386 |
| Acsl4 | NM_001033600 | acyl-CoA synthetase long-chain family member 4 | 2.83 | 0.0003014 |
| A030012M09Rik | NM_183028 | RIKEN cDNA A030012M09 gene | 2.83 | 0.0003146 |
| Rbpsuh | NM_009035 | Recombining binding protein suppressor of hairless (Drosophila) | 2.83 | 0.00054124 |
| Acvr2a | NM_007396 | Activin receptor IIA | 2.85 | 0.00011208 |
| Sp3 | NM_011450 | trans-acting transcription factor 3 | 2.85 | 0.01371137 |
| Gna13 | NM_010303 | Guanine nucleotide binding protein, alpha 13 (Gna13), mRNA | 2.85 | 0.00108932 |
| Bclaf1 | NM_001025392 | BCL2-associated transcription factor 1 | 2.85 | 0.00125007 |
| Crk | NM_133656 | v-crk sarcoma virus CT10 oncogene homolog (avian) | 2.86 | 0.00315405 |
| Itga4 | NM_010576 | Integrin alpha 4 | 2.86 | 0.00740739 |
| Calu | NM_007594 | calumenin | 2.87 | 0.00056605 |
| Pja2 | NM_001025309 | Praja 2, RING-H2 motif containing (Pja2), transcript variant 2, mRNA | 2.87 | 0.0014305 |
| Ccl17 | NM_011332 | chemokine (C-C motif) ligand 17 | 2.88 | 0.00212358 |
| Ptk9 | NM_008971 | protein tyrosine kinase 9 | 2.88 | 0.00039293 |
| 4930429H24Rik | NM_029436 | RIKEN cDNA 4930429H24 gene | 2.88 | 0.00971094 |
| Flnb | XM_127565 | Filamin, beta, mRNA | 2.90 | 0.00042715 |
| 2610005L07Rik | XM_486329 | RIKEN cDNA 2610005L07 gene | 2.90 | 0.00137795 |
| Abca1 | NM_013454 | ATP-binding cassette, sub-family A (ABC1), member 1 | 2.92 | 0.00046641 |
| 5730454B08Rik | NM_144530 | Zinc finger CCCH type containing 11A (Zc3h11a), mRNA | 2.93 | 0.00023486 |
| 3830408G10Rik | XM_355676 | RIKEN cDNA 3830408G10 gene | 2.93 | 0.00067968 |
| Kitl | NM_013598 | Kit ligand | 2.93 | 0.02345994 |
| Vps35 | NM_022997 | vacuolar protein sorting 35 | 2.93 | 0.00460622 |
| Mme | NM_008604 | membrane metallo endopeptidase | 2.94 | 0.00097667 |
| Srrm2 | NM_175229 | serine/arginine repetitive matrix 2 | 2.94 | 0.00064775 |
| Adam17 | NM_009615 | a disintegrin and metallopeptidase domain 17 | 2.95 | 0.00124342 |
| Rrm2b | NM_199476 | ribonucleotide reductase M2 B (TP53 inducible) | 2.96 | 0.00013855 |
| Rapgef2 | XM_203999 | Rap guanine nucleotide exchange factor (GEF) 2, mRNA | 2.97 | 0.00088512 |
| 2300006M17Rik | --- | RIKEN cDNA 2010111I01 gene, mRNA | 2.97 | 0.00062468 |
| Arid4b | NM_194262 | AT rich interactive domain 4B (Rbp1 like) | 2.98 | 0.00514524 |
| Msrb3 | NM_177092 | Methionine sulfoxide reductase B3 | 2.99 | 0.01657352 |
| Tes3-ps | NM_033623 | testis derived transcript 3, pseudogene | 3.00 | 0.0015219 |
| Ceacam1 | NM_011926 | CEA-related cell adhesion molecule 1 | 3.00 | 0.00930931 |
| Rnf12 | NM_011276 | Ring finger protein 12, mRNA | 3.01 | 0.00083791 |
| Tgtp | NM_011579 | T-cell specific GTPase | 3.01 | 0.00065246 |
| 2810436B12Rik | --- | RIKEN cDNA 2810436B12 gene | 3.01 | 0.00055158 |
| Klrb1d | NM_030599 | killer cell lectin-like receptor subfamily B member 1D | 3.02 | 0.00807271 |
| Rbm5 | NM_148930 | RNA binding motif protein 5 | 3.02 | 0.00015138 |
| 2810013E07Rik | NM_178112 | RIKEN cDNA 2810013E07 gene | 3.02 | 0.00114735 |
| A630082K20Rik | XM_145254 | RIKEN cDNA A630082K20 gene | 3.02 | 0.00184446 |
| Usp47 | NM_133758 | ubiquitin specific peptidase 47 | 3.03 | 0.00016566 |
| Ahcyl1 | NM_145542 | S-adenosylhomocysteine hydrolase-like 1 | 3.03 | 0.00129709 |
| Ncf1 | NM_010876 | neutrophil cytosolic factor 1 | 3.03 | 0.00415926 |
| Rlf | XM_355515 | Rearranged L-myc fusion sequence, mRNA | 3.04 | 0.0000955 |
| Mast4 | XM_283179 | microtubule associated serine/threonine kinase family member 4 | 3.04 | 0.00010213 |
| Itgam | NM_008401 | integrin alpha M | 3.05 | 0.0000703 |
| AI426953 | --- | expressed sequence AI426953 | 3.07 | 0.00105674 |
| Pik3c2a | NM_011083 | phosphatidylinositol 3-kinase, C2 domain containing, alpha polypeptide | 3.08 | 0.00763764 |
| Mpa2l | NM_194336 | macrophage activation 2 like | 3.09 | 0.00058818 |
| Pkp2 | NM_026163 | Plakophilin 2 (Pkp2), mRNA | 3.09 | 0.00107513 |
| Sqstm1 | NM_011018 | Sequestosome 1 | 3.11 | 0.00046054 |
| Gpiap1 | NM_016739 | GPI-anchored membrane protein 1 | 3.12 | 0.00138812 |
| Whsc1l1 | XM_620640 | Wolf-Hirschhorn syndrome candidate 1-like 1 (human) | 3.14 | 0.0000813 |
| 2810403A07Rik | NM_028814 | RIKEN cDNA 2810403A07 gene (2810403A07Rik), mRNA | 3.14 | 0.0002068 |
| Evi2b | NM_146023 | ecotropic viral integration site 2b | 3.14 | 0.00130903 |
| Jak1 | NM_146145 | Janus kinase 1 | 3.14 | 0.00016606 |
| Dcun1d1 | NM_033623 | defective in cullin neddylation 1, domain containing 1 (S. cerevisiae) | 3.14 | 0.00026777 |
| Ube3a | NM_001033962 | Ubiquitin protein ligase E3A | 3.15 | 0.0000748 |
| Rps6ka3 | NM_148945 | ribosomal protein S6 kinase polypeptide 3 | 3.15 | 0.01451882 |
| R3hdm | NM_181750 | R3H domain (binds single-stranded nucleic acids), mRNA | 3.15 | 0.00063977 |
| 5730416O20Rik | --- | RIKEN cDNA 5730416O20 gene | 3.16 | 0.0000709 |
| Ccl9 | NM_011338 | chemokine (C-C motif) ligand 9 | 3.16 | 0.00341445 |
| 0610037M15Rik | --- | RIKEN cDNA 0610037M15 gene | 3.17 | 0.00041573 |
| Picalm | NM_146194 | Phosphatidylinositol binding clathrin assembly protein, mRNA | 3.18 | 0.00019117 |
| Ppp3ca | NM_008913 | Protein phosphatase 3, catalytic subunit, alpha isoform (Ppp3ca), mRNA | 3.18 | 0.0000768 |
| Cxcl13 | NM_018866 | chemokine (C-X-C motif) ligand 13 | 3.19 | 0.01121365 |
| Birc4 | NM_009688 | Baculoviral IAP repeat-containing 4 (Birc4), mRNA | 3.20 | 0.00050179 |
| Aplp2 | NM_009691 | amyloid beta (A4) precursor-like protein 2 | 3.21 | 0.0000975 |
| Etv3 | NM_012051 | ets variant gene 3 | 3.22 | 0.0000932 |
| Pard3 | NM_001013580 | partitioning defective 3 homolog (C. elegans), transcript variant 3, mRNA | 3.22 | 0.00039158 |
| Fbxw11 | NM_134015 | F-box and WD-40 domain protein 11 | 3.22 | 0.00362483 |
| 5530401D11Rik | --- | RIKEN cDNA 5530401D11 gene | 3.23 | 0.000068 |
| Man1a | NM_008548 | mannosidase 1, alpha | 3.23 | 0.01570362 |
| Tank | NM_011529 | TRAF family member-associated Nf-kappa B activator, mRNA | 3.24 | 0.0000828 |
| Scfd1 | NM_029825 | sec1 family domain containing 1 | 3.25 | 0.0006621 |
| Tloc1 | NM_027016 | Translocation protein 1 | 3.28 | 0.00950111 |
| 1200009B18Rik | NM_026168 | RIKEN cDNA 1200009B18 gene | 3.28 | 0.00076345 |
| D5Ertd798e | --- | DNA segment, Chr 5, ERATO Doi 798, expressed | 3.29 | 0.00094141 |
| Tbl1x | NM_020601 | transducin (beta)-like 1 X-linked | 3.30 | 0.00658492 |
| Stk17b | NM_133810 | serine/threonine kinase 17b (apoptosis-inducing) | 3.30 | 0.00740901 |
| Gspt1 | NM_146066 | G1 to S phase transition 1 | 3.31 | 0.00019464 |
| 2410042D21Rik | NM_024254 | RIKEN cDNA 2410042D21 gene | 3.31 | 0.00037276 |
| Camk2d | NM_001025438 | calcium/calmodulin-dependent protein kinase II, delta | 3.31 | 0.00020846 |
| Lmo4 | NM_010723 | LIM domain only 4, mRNA | 3.32 | 0.0000965 |
| 9130221J17Rik | --- | RIKEN cDNA 9130221J17 gene | 3.34 | 0.00031473 |
| Fubp1 | NM_057172 | Far upstream element (FUSE) binding protein 1 (Fubp1), mRNA | 3.34 | 0.00084756 |
| C3ar1 | NM_009779 | complement component 3a receptor 1 | 3.34 | 0.0010948 |
| Ywhae | NM_009536 | Tyrosine 3-monooxygenase/tryptophan 5-monooxygenase activation protein, epsilon polypeptide | 3.34 | 0.0000489 |
| Trps1 | NM_032000 | trichorhinophalangeal syndrome I (human) | 3.35 | 0.00045746 |
| AU015680 | --- | expressed sequence AU015680 | 3.36 | 0.00061083 |
| Nmd3 | NM_133787 | NMD3 homolog (S. cerevisiae) | 3.36 | 0.00069396 |
| Fbxo30 | NM_027968 | F-box protein 30 | 3.37 | 0.00141018 |
| Fancc | NM_007985 | Fanconi anemia, complementation group C | 3.37 | 0.00062468 |
| Plek | NM_019549 | pleckstrin | 3.37 | 0.0025709 |
| A930007B11Rik | --- | RIKEN cDNA A930007B11 gene | 3.39 | 0.00065181 |
| 4933426M11Rik | NM_178682 | MKIAA0247 protein | 3.39 | 0.0000715 |
| Cxcr6 | NM_030712 | chemokine (C-X-C motif) receptor 6 | 3.39 | 0.00731587 |
| Pi4k2b | NM_025951 | phosphatidylinositol 4-kinase type 2 beta | 3.42 | 0.02356982 |
| Tde2 | NM_019760 | tumor differentially expressed 2 | 3.45 | 0.00729825 |
| Epm2a | NM_010146 | epilepsy, progressive myoclonic epilepsy, type 2 gene alpha | 3.47 | 0.0000609 |
| Il7r | NM_008372 | interleukin 7 receptor | 3.47 | 0.00715147 |
| Cybb | NM_007807 | cytochrome b-245, beta polypeptide | 3.48 | 0.00625389 |
| Esd | NM_016903 | Esterase D/formylglutathione hydrolase (Esd), mRNA | 3.49 | 0.00011808 |
| LOC553096 | --- | hypothetical LOC553096 | 3.50 | 0.0000815 |
| 9130208E07Rik | --- | RIKEN cDNA 9130208E07 gene | 3.51 | 0.00028691 |
| Ewsr1 | NM_007968 | Ewing sarcoma breakpoint region 1 (Ewsr1), mRNA | 3.51 | 0.0001965 |
| Rab18 | NM_011225 | RAB18, member RAS oncogene family | 3.52 | 0.0007139 |
| 2900045N06Rik | NM_028385 | RIKEN cDNA 2900045N06 gene, mRNA | 3.53 | 0.00042773 |
| 5830411E10Rik | NM_028696 | RIKEN cDNA 5830411E10 gene | 3.56 | 0.00429517 |
| Trem1 | NM_021406 | triggering receptor expressed on myeloid cells 1 | 3.60 | 0.00034084 |
| Cask | NM_009806 | calcium/calmodulin-dependent serine protein kinase (MAGUK family) | 3.60 | 0.00126284 |
| E230012J19Rik | --- | RIKEN cDNA E230012J19 gene | 3.60 | 0.00026004 |
| LOC553089 | --- | hypothetical LOC553089 | 3.61 | 0.0000669 |
| Igsf6 | NM_030691 | immunoglobulin superfamily, member 6 | 3.61 | 0.00081201 |
| 2610005L07Rik | XM_486359 | RIKEN cDNA 2610005L07 gene | 3.64 | 0.00301466 |
| Rab14 | NM_026697 | RAB14, member RAS oncogene family | 3.65 | 0.00040275 |
| Slc39a10 | NM_172653 | solute carrier family 39 (zinc transporter), member 10 | 3.66 | 0.00050298 |
| Gdi3 | NM_008112 | Guanosine diphosphate (GDP) dissociation inhibitor 3 | 3.67 | 0.00172388 |
| Diap1 | NM_007858 | Diaphanous homolog 1 (Drosophila) (Diap1), mRNA | 3.70 | 0.00023075 |
| Eif4e | NM_007917 | Eukaryotic translation initiation factor 4E, mRNA | 3.71 | 0.00016121 |
| A230061C15Rik | --- | RIKEN cDNA A230061C15 gene | 3.73 | 0.00020755 |
| 5830417C01Rik | NM_024282 | RIKEN cDNA 5830417C01 gene (5830417C01Rik), mRNA | 3.73 | 0.00074419 |
| Pabpc1 | NM_008774 | poly A binding protein, cytoplasmic 1 | 3.76 | 0.0000606 |
| 9630026M06Rik |  | RIKEN cDNA 9630026M06 gene | 3.78 | 0.00056343 |
| AW011752 | NM_134034 | expressed sequence AW011752 | 3.79 | 0.00119105 |
| Nedd4l | NM_031881 | neural precursor cell expressed, developmentally down-regulated gene 4-like | 3.79 | 0.0000693 |
| Atf2 | NM_001025093 | Activating transcription factor 2 | 3.82 | 0.00277332 |
| Sacm1l | NM_030692 | SAC1 (supressor of actin mutations 1, homolog)-like (S. cerevisiae), mRNA | 3.83 | 0.00073943 |
| Rap1b | NM_024457 | RAS related protein 1b | 3.89 | 0.00969644 |
| 8430417A20Rik | NM_175209 | RIKEN cDNA 8430417A20 gene | 3.93 | 0.0000772 |
| Kcnq1ot1 | NR_001461 | KCNQ1 overlapping transcript 1 | 3.94 | 0.00012675 |
| Cstf3 | NM_145529 | Cleavage stimulation factor, 3' pre-RNA, subunit 3 (Cstf3), mRNA | 3.96 | 0.0000408 |
| Ctsc | NM_009982 | Cathepsin C | 3.97 | 0.00361295 |
| Mbtd1 | NM_134012 | mbt domain containing 1 | 3.98 | 0.00066611 |
| Nbeal1 | --- | Neurobeachin like 1, mRNA | 3.99 | 0.00105215 |
| Cdh1 | NM_009864 | Cadherin 1 (Cdh1), mRNA | 4.01 | 0.00067197 |
| Ptdsr | NM_033398 | RIKEN cDNA 1110005A03 gene (1110005A03Rik), mRNA | 4.01 | 0.00031506 |
| 4833441D16Rik | --- | RIKEN cDNA 4833441D16 gene | 4.01 | 0.00059629 |
| Syk | NM_011518 | spleen tyrosine kinase | 4.02 | 0.00139056 |
| 4833416J08Rik | --- | RIKEN cDNA 4833416J08 gene | 4.04 | 0.0002688 |
| Sca1 | NM_009124 | Spinocerebellar ataxia 1 homolog (human) (Sca1), mRNA | 4.09 | 0.0000633 |
| Chka | NM_013490 | choline kinase alpha | 4.09 | 0.00231612 |
| Arid5b | NM_023598 | Modulator recognition factor 2 (Mrf2) | 4.09 | 0.00100212 |
| Nfat5 | NM_018823 | Nuclear factor of activated T-cells 5 (Nfat5) | 4.11 | 0.00014689 |
| Sfrs11 | NM_026989 | splicing factor, arginine/serine-rich 11 | 4.13 | 0.0000801 |
| Jmjd1c | XM_354543 | MKIAA1380 protein | 4.13 | 0.00019572 |
| D930017J03Rik | --- | RIKEN cDNA D930017J03 gene | 4.14 | 0.000067 |
| 5330406M23Rik | --- | RIKEN cDNA 5330406M23 gene | 4.16 | 0.00077957 |
| Ahctf1 | NM_026375 | AT hook containing transciption factor 1 | 4.18 | 0.000142 |
| Met | NM_008591 | met proto-oncogene | 4.18 | 0.0018915 |
| Ms4a6b | NM_027209 | Membrane-spanning 4-domains, subfamily A, member 6B, mRNA | 4.21 | 0.00038011 |
| 2310032D16Rik | NM_027096 | RIKEN cDNA 2310032D16 gene | 4.23 | 0.00033362 |
| A430108E01Rik | --- | RIKEN cDNA A430108E01 gene | 4.23 | 0.00028891 |
| A430102J17Rik | --- | RIKEN cDNA A430102J17 gene | 4.25 | 0.00025681 |
| Lgals3 | NM_010705 | Lectin, galactose binding, soluble 3 (Lgals3), mRNA | 4.28 | 0.00087632 |
| Osbpl9 | NM_133885 | Oxysterol binding protein-like 9 | 4.28 | 0.00031271 |
| Ranbp2 | NM_011240 | RAN binding protein 2 | 4.29 | 0.00037422 |
| Jarid2 | NM_021878 | Jumonji, AT rich interactive domain 2, mRNA | 4.36 | 0.00137393 |
| Mbnl2 | NM_175341 | MKIAA4072 protein | 4.37 | 0.00129209 |
| 5730526G10Rik |  | RIKEN cDNA 5730526G10 gene | 4.42 | 0.00042937 |
| 9530029O12Rik | --- | RIKEN cDNA 9530029O12 gene | 4.42 | 0.00010719 |
| Sdfr1 | NM_009145 | Stromal cell derived factor receptor 1 (Sdfr1), mRNA | 4.43 | 0.00172525 |
| H3f3b | NM_008211 | H3 histone, family 3B | 4.45 | 0.00013554 |
| A530088I07Rik | NM_175437 | RIKEN cDNA A530088I07 gene | 4.51 | 0.00109321 |
| D9Ertd306e | --- | DNA segment, Chr 9, ERATO Doi 306, expressed | 4.51 | 0.0000989 |
| Fgl2 | NM_008013 | fibrinogen-like protein 2 | 4.54 | 0.00190913 |
| Axot | NM_020575 | Axotrophin | 4.56 | 0.00634256 |
| 1110001A05Rik | NM_019808 | RIKEN cDNA 1110001A05 gene | 4.56 | 0.00861753 |
| A430061O12Rik | --- | RIKEN cDNA A430061O12 gene | 4.61 | 0.00032391 |
| BC023969 | --- | cDNA sequence BC023969 | 4.63 | 0.00101071 |
| Mtac2d1 | NM_028924 | membrane targeting (tandem) C2 domain containing 1 | 4.63 | 0.00434247 |
| AI848100 | XM_484932 | expressed sequence AI848100 | 4.67 | 0.00012616 |
| Hif1a | NM_010431 | hypoxia inducible factor 1, alpha subunit | 4.69 | 0.0000429 |
| Mapk8 | NM_016700 | mitogen activated protein kinase 8 | 4.70 | 0.00182178 |
| Copa | NM_009938 | coatomer protein complex subunit alpha | 4.71 | 0.00108801 |
| A430108E01Rik | NM_001033637 | RIKEN cDNA A430108E01 gene | 4.74 | 0.00056352 |
| Rtn4* | NM_024226 | reticulon 4 | 4.86 | 0.0000254 |
| Klf5 | NM_009769 | Kruppel-like factor 5 | 4.89 | 0.00037509 |
| 9430063L05Rik | NM_178080 | RIKEN cDNA 9430063L05 gene | 4.92 | 0.00030362 |
| S100a8 | NM_013650 | S100 calcium binding protein A8 (calgranulin A) | 4.96 | 0.00827169 |
| Cap1 | NM_007598 | CAP, adenylate cyclase-associated protein 1 (yeast) | 4.99 | 0.00264674 |
| 5830411K21Rik | --- | RIKEN cDNA 5830411K21 gene | 5.05 | 0.00058015 |
| Gmfb | NM_022023 | glia maturation factor, beta | 5.07 | 0.00051216 |
| Nr3c1 | NM_008173 | nuclear receptor subfamily 3, group C, member 1 | 5.09 | 0.00092345 |
| Ddx3y | NM_012008 | DEAD (Asp-Glu-Ala-Asp) box polypeptide 3, Y-linked | 5.11 | 0.0000212 |
| Wdr26 | --- | WD repeat domain 26 | 5.24 | 0.00641978 |
| Pdlim5 | NM_019808 | PDZ and LIM domain 5, mRNA | 5.25 | 0.00394351 |
| A730009E18Rik | --- | RIKEN cDNA A730009E18 gene | 5.27 | 0.00032218 |
| Erbb2ip | NM_001005868 | Erbb2 interacting protein | 5.31 | 0.00937821 |
| Iigp1 | NM_021792 | interferon inducible GTPase 1 | 5.35 | 0.0000136 |
| B930025B16Rik | --- | RIKEN cDNA B930025B16 gene | 5.39 | 0.0000199 |
| Alcam | NM_009655 | activated leukocyte cell adhesion molecule | 5.43 | 0.00014325 |
| Gdap10 | --- | ganglioside-induced differentiation-associated-protein 10 | 5.46 | 0.0000226 |
| S100a9 | NM_009114 | S100 calcium binding protein A9 (calgranulin B) | 5.53 | 0.00411439 |
| Mlstd2 | NM_026143 | male sterility domain containing 2 | 5.54 | 0.00139437 |
| Pde4b | NM_019840 | phosphodiesterase 4B, cAMP specific | 5.58 | 0.00388738 |
| Ptgs2 | NM_011198 | prostaglandin-endoperoxide synthase 2 | 5.69 | 0.00887498 |
| Serpinb6a | NM_009254 | Serine (or cysteine) peptidase inhibitor, clade B, member 6a, mRNA | 5.70 | 0.00290446 |
| 1500010G04Rik | NM_173366 | RIKEN cDNA 1500010G04 gene | 5.85 | 0.0000721 |
| AU015263 | --- | expressed sequence AU015263 | 5.86 | 0.0000723 |
| Strn3 | NM_052973 | Striatin, calmodulin binding protein 3 (Strn3), mRNA | 5.95 | 0.00062987 |
| Luc7l2 | NM_138680 | LUC7-like 2 (S. cerevisiae) | 6.01 | 0.00454217 |
| Cnbp1 | NM_013493 | Cellular nucleic acid binding protein 1 (Cnbp1), mRNA | 6.09 | 0.00044148 |
| Rybp | NM_019743 | RING1 and YY1 binding protein (Rybp), mRNA | 6.25 | 0.0000637 |
| Malt1* | NM_172833 | Mucosa associated lymphoid tissue lymphoma translocation gene 1, mRNA | 6.27 | 0.00000963 |
| 1700034P14Rik | NM_028487 | GC-rich promoter binding protein 1, mRNA | 6.42 | 0.00032422 |
| Rnpc2 | NM_133242 | RNA-binding region (RNP1, RRM) containing 2, mRNA | 6.44 | 0.00128221 |
| Tug1 | NR_002321 | taurine upregulated gene 1 | 6.61 | 0.00050682 |
| C430003N24Rik | --- | RIKEN cDNA C430003N24 gene | 7.09 | 0.00000996 |
| Gpbp1 | NM_028487 | GC-rich promoter binding protein 1 | 7.17 | 0.00508434 |
| Cblb | XM_156257 | Casitas B-lineage lymphoma b | 7.29 | 0.00099798 |
| C79248 | NM_133242 | expressed sequence C79248 | 7.40 | 0.00480671 |
| 2010010M04Rik | --- | RIKEN cDNA 2010010M04 gene | 7.55 | 0.00012356 |
| Ptbp2 | NM_019550 | Polypyrimidine tract binding protein 2 | 8.08 | 0.01311114 |
| Gls | XM_129846 | MKIAA0838 protein | 8.14 | 0.0076447 |
| Adk | NM_134079 | adenosine kinase | 8.32 | 0.0000347 |
| 5830407P18Rik | --- | RIKEN cDNA 5830407P18 gene | 8.37 | 0.00000683 |
| Eif2s2 | NM_026030 | eukaryotic translation initiation factor 2, subunit 2 (beta) | 9.75 | 0.00000537 |
| BC023105 | NM_145357 | cDNA sequence BC023105 | 13.66 | 0.00024213 |
|  |  |  |  |  |
|  |  |  |  |  |
| Genes labeled with * were verified by RT-PCR, western blot or Immunostaing. | | |  |  |


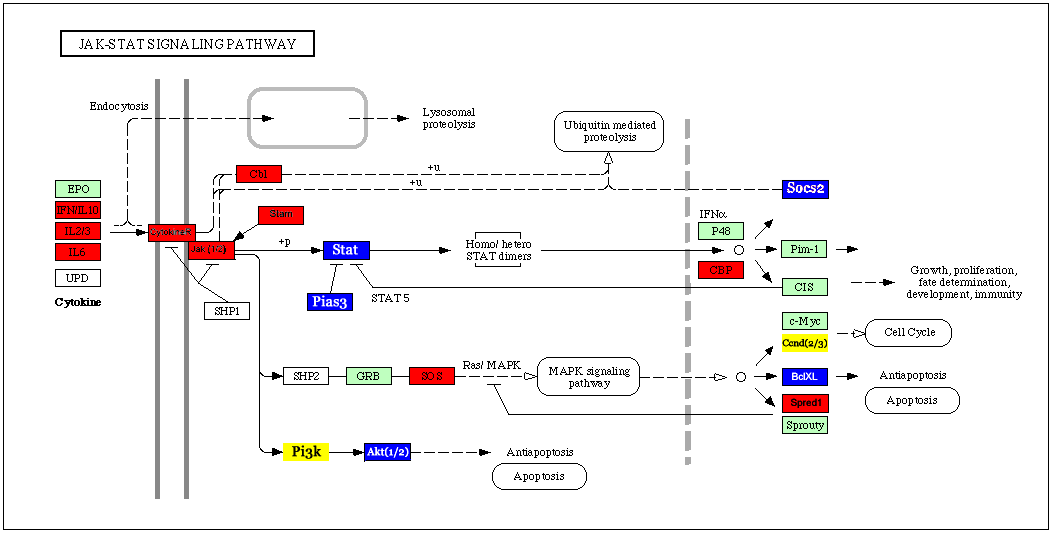


Supplementary Figures 1


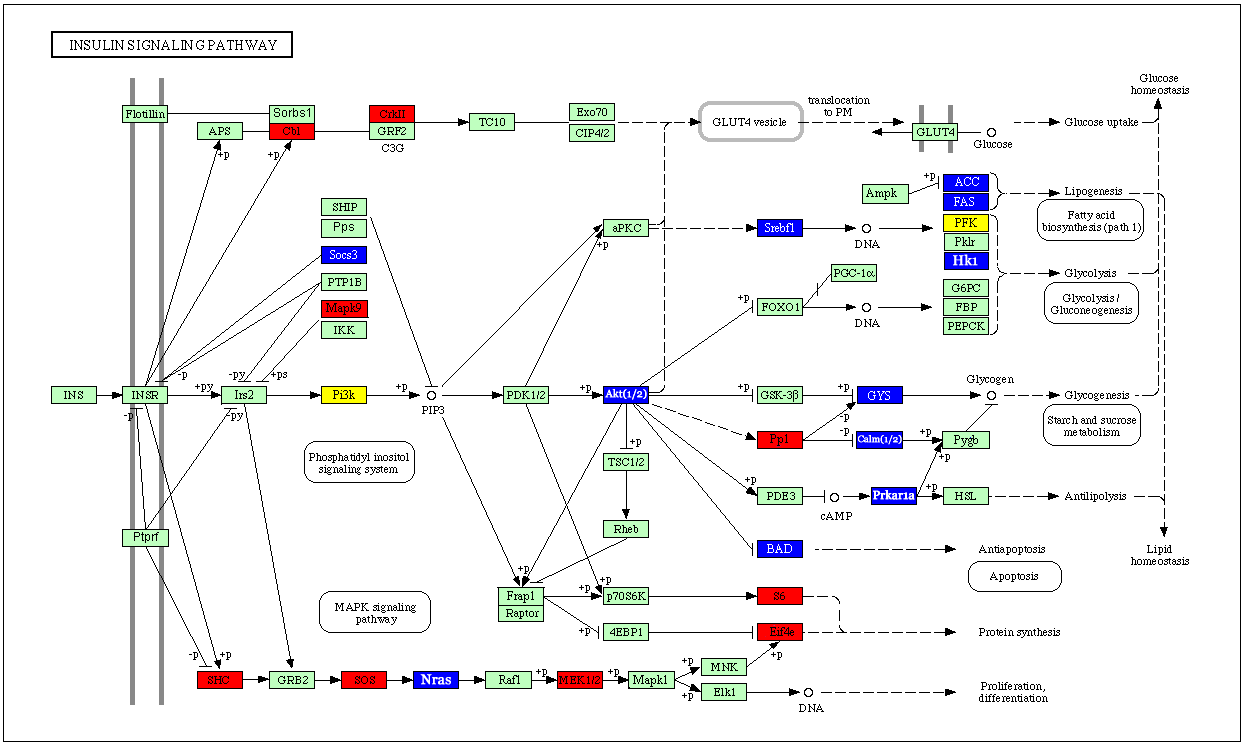


Supplementary Figures 2


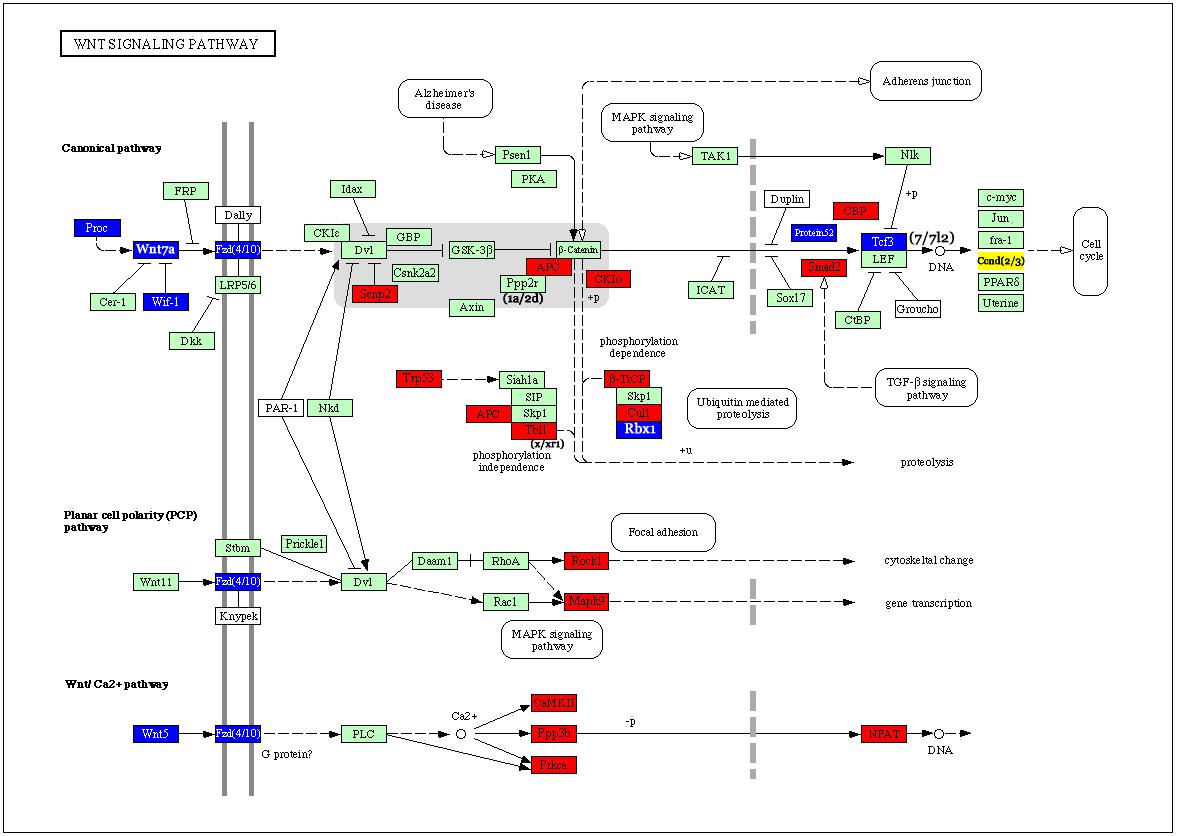


Supplementary Figures 3
