# Supplementary figures and images for: Gene expression and biological processes influenced by deletion of Stat3 in pulmonary type II epithelial cells
Source: BMC Genomics. 2007 Dec 10;8:455. doi: 10.1186/1471-2164-8-455 (PMC2234434; doi:10.1186/1471-2164-8-455)

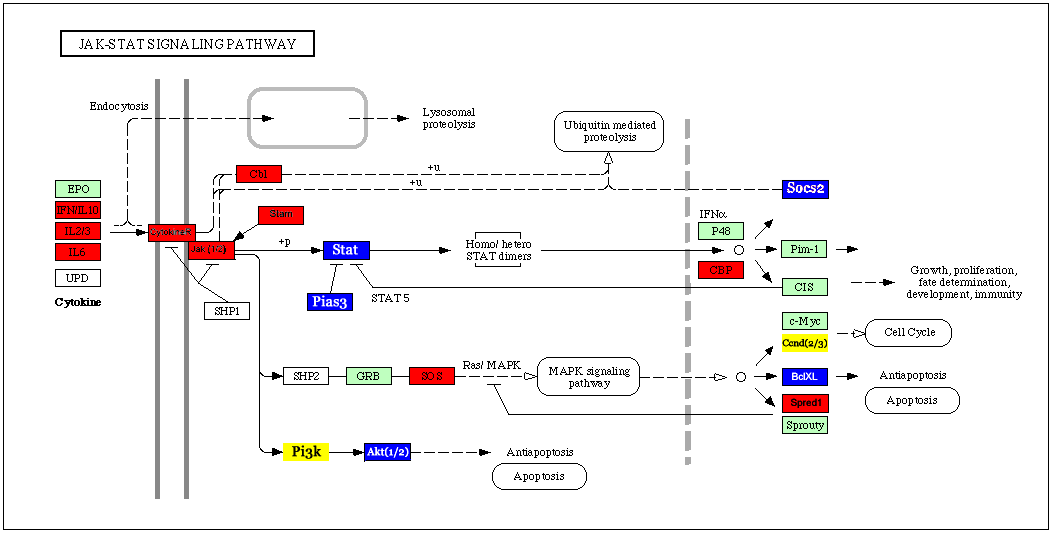


Supplementary Figures 1


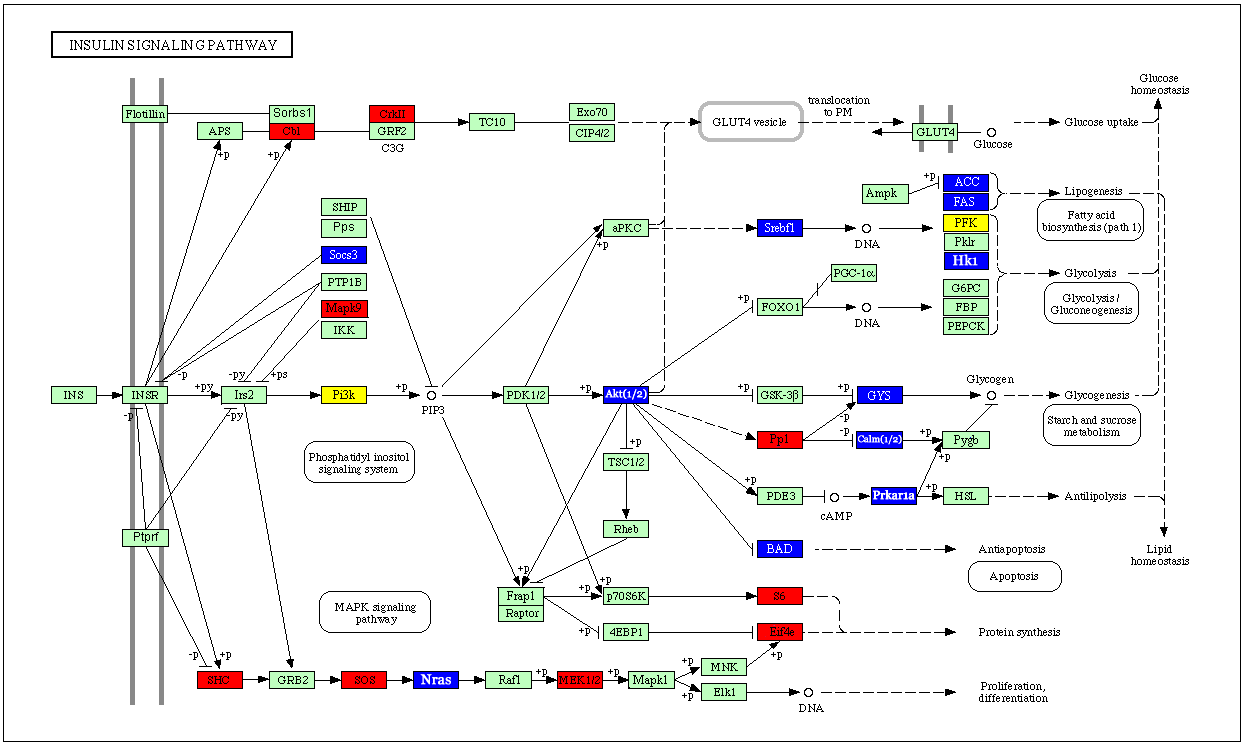


Supplementary Figures 2


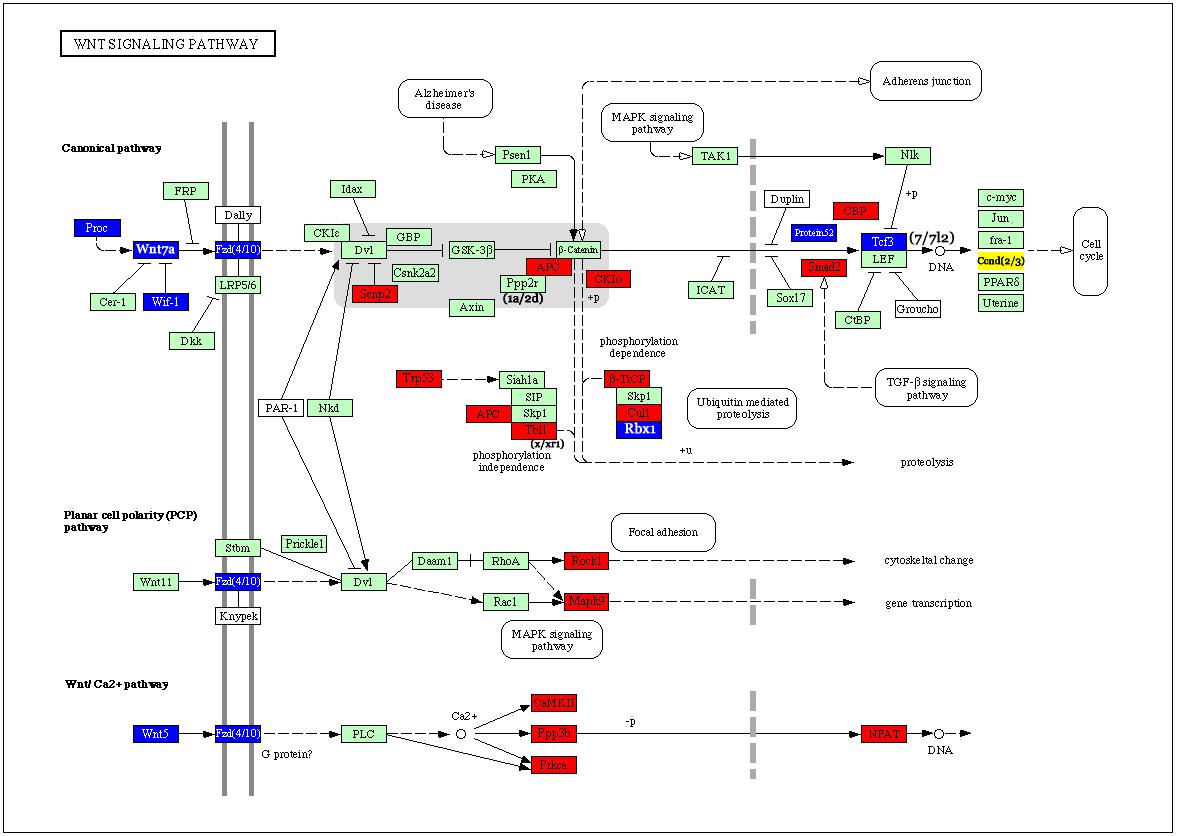


Supplementary Figures 3

Supplement: Additional file 2 — Differentially Expressed Genes In Jak-Stat, Insulin and Wnt Signaling Pathway. Pathways were downloaded from KEGG website [82]. Each rectangle represents one or multiple gene products. Genes up-regulated in Stat3Δ/Δ mice are highlighted in red. Genes down-regulated in Stat3Δ/Δ mice are highlighted in blue. Rectangle contains more than one gene products and changes expression in opposite directions are highlighted in yellow. [file 1471-2164-8-455-S2.doc]
